# Mesenchymal stem cells delivered via a bioactive disordered peptide-hydrogel platform modulate early inflammation and enhance skeletal repair in a polytrauma model

**DOI:** 10.1177/20417314251397106

**Published:** 2025-12-01

**Authors:** Augustine Mark Saiz, Maryam Rahmati, Tony Daniel Baldini, Aneesh Satish Bhat, Soren David Johnson, Mengyao Liu, Renato Miguel Reyes, Shierly W. Fok, Mark A. Lee, Thaqif El Khassawna, D. C. Florian Wieland, André Lopes Marinho, Clement Blanchet, J. Kent Leach, Håvard Jostein Haugen

**Affiliations:** 1 Department of Orthopaedic Surgery, UC Davis Health, Sacramento, CA, USA; 2 Department of Biomaterials, Institute of Clinical Dentistry, University of Oslo, Norway; 3 California Northstate University College of Medicine, Elk Grove, USA; 4 Department of Biomedical Engineering, UC Davis, CA, USA; 5 Experimental Trauma Surgery, Justus-Liebig University Giessen, Germany; 6 School of Pharmacy, The University of Jordan, Amman, Jordan; 7 Institute of Metallic Biomaterials, Helmholtz Zentrum Hereon, Geesthacht, Germany; 8 European Molecular Biology Laboratory EMBL, Hamburg Site, Germany

**Keywords:** intrinsically disordered peptides, hyaluronic acid, hydrogel, inflammation, polytrauma

## Abstract

Over 30% of polytrauma patients with bone fractures suffer from impaired healing and nonunion due to persistent systemic inflammation. Existing biologic strategies for bone repair primarily focus on osteogenesis but are not designed to modulate systemic immune dysregulation, limiting their utility in the polytrauma setting. To overcome this, we developed a hyaluronic acid-based hydrogel (HA) incorporating osteogenic intrinsically disordered peptides (P2) and mesenchymal stem cells (MSCs) to promote bone regeneration and modulate inflammation simultaneously. MSCs entrapped in hydrogels containing P2 (HA + P2) exhibited increased cell viability, alkaline phosphatase activity, and calcium deposition under in vitro polytrauma conditions compared to MSCs in hydrogels alone (HA). We utilized a murine polytrauma model (4 mm femoral osteotomy + blunt chest trauma) in mice. We studied the inflammatory response and bone formation over 21 days in mice treated with (1) HA, (2) HA + P2, or (3) HA + P2 + MSCs. We observed that adding P2 enhanced bone mineralization at the fracture site, yet transplantation of MSCs with P2 further increased mineralization. Both HA + P2 and HA + P2 + MSCs groups attenuated the systemic inflammatory response to near healthy baseline values. The HA + P2 group significantly accelerated the first stages of fracture healing by upregulating genes encoding for collagen biosynthesis, modifying enzymes, and extracellular matrix (ECM)-receptor interaction. Mice treated with HA + P2 + MSCs exhibited transcriptional regulation resulting in the upregulation of key repair genes related to cell cycle control, E2F transcriptional regulation, and TP53-mediated DNA repair, alongside downregulation of inflammatory pathways (IL-2, IL-3, and IL-5 signaling) and improved fracture healing. This study demonstrated that the combination of intrinsically disordered peptides and mesenchymal stem cells in HA-based hydrogels enhances bone formation, modulates both local and systemic inflammation, and improves structural organization at the fracture site in polytrauma conditions.

## Introduction

Bone tissue is a dynamic and complex structure that provides mechanical support and protection for vital organs and plays a crucial role in hematopoiesis and mineral homeostasis.^1,2^ Bone consists of a mineralized extracellular matrix (ECM) composed primarily of collagen and non-collagenous proteins that regulate crystal nucleation and growth during bone mineralization.^3^ These interactions are essential for maintaining bone integrity and function. Among the proteins involved, intrinsically disordered proteins (IDPs) have gained significant attention due to their unique structural and biochemical properties.^4^ IDPs lack a fixed three-dimensional structure, allowing them to adapt to various biochemical environments.^5^ This flexibility enables IDPs to participate in various molecular and cellular signaling pathways, making them critical players in bone biomineralization.^6,7^ Their role in modulating protein-mineral and protein-protein interactions highlights their potential use in regenerative medicine, particularly in inducing and/or enhancing osteoinductive and osteoconductive properties in biomaterials such as bone grafts and calcium phosphate-based scaffolds.^4,6,7^

Fracture healing involves tightly coordinated inflammatory, reparative, and remodeling phases.^8^ Following injury, immune activation initiates cellular recruitment and tissue repair, ultimately forming a mineralized fracture callus. In polytrauma, where patients suffer multiple injuries including fractures, soft tissue damage, and internal organ trauma, this process is severely disrupted by a prolonged, dysregulated inflammatory response.^9–11^ Persistently elevated cytokines and leukocyte infiltration interfere with callus formation, resulting in impaired mineralization and delayed healing. Our previous work using a murine model combining blunt femur fracture and chest trauma, alongside data from other studies, demonstrated these effects.^10,12–14^ Despite advancements in surgical techniques and postoperative care, therapeutic options for managing fracture healing in polytrauma patients remain limited. While current biologic materials, such as bone morphogenetic proteins (BMPs) on collagen sponges or demineralized-based matrix (DBM)-based products, are primarily utilized in nonunion repair, these strategies are not designed to address the systemic immune dysregulation observed in polytrauma. This limitation reduces their effectiveness in acute multisystem injury settings, where a prolonged inflammatory response can impede bone healing. For instance, studies have indicated that BMP-2 delivered via collagen sponges may correlate with systemic immune dysregulation, highlighting the need for biomaterials that can modulate the immune response in addition to promoting osteogenesis.^15^ Therefore, innovative approaches that address both the inflammatory and osteogenic components of healing are needed.

Hyaluronic acid (HA)-based hydrogels offer a promising platform for addressing these challenges. HA is a naturally occurring glycosaminoglycan that provides three critical functions in our therapeutic system: (1) Cell delivery and retention: HA creates a hydrophilic, biocompatible microenvironment that maintains MSC viability and prevents rapid cell clearance, enabling sustained cellular therapeutic effects at the fracture site, as demonstrated in our recent publication^16^; (2) Controlled biomolecule release: HA’s hydrogel network enables sustained, controlled release of therapeutic peptides, preventing the burst release and rapid clearance that occurs with direct injection while maintaining prolonged bioavailability; and (3) Immunomodulatory scaffold properties: HA exhibits intrinsic anti-inflammatory effects by reducing excessive macrophage M1 polarization and promoting M2 phenotype transition, complementing MSC-mediated immunomodulation.^17^ However, the intrinsic osteoinductivity and osteoconductivity of HA is limited, requiring bioactive enhancement for effective bone regeneration.^18^ Our recent work demonstrated that while MSCs delivered locally in hyaluronic acid hydrogels successfully reduced inflammation in polytrauma, they failed to achieve complete fracture healing within 3 weeks, highlighting the need for osteoinductive augmentation. To address this gap, we created a multifunctional osteo-immunomodulatory system by incorporating the proline-rich IDP P2 and MSCs into the HA hydrogel platform. HA serves as the essential delivery vehicle that enables both components to function: it retains MSCs at the injury site for sustained immunomodulation and provides controlled P2 release for prolonged osteoinduction. We selected P2 to enhance the bone-forming potential of the HA-MSC platform because this peptide mimics the function of non-collagenous ECM proteins by modulating mineral nucleation and promoting sustained osteoblast differentiation.^4,19,20^ Unlike short-acting commercial additives, such as covalently linked peptides like the BMP-2 knuckle epitope, which often require advanced delivery systems due to rapid diffusion and limited bioactivity, P2 maintains sustained osteogenic activity.^4,21^ This is attributed to its intrinsically disordered structure, which mimics the dynamic function of native ECM proteins and promotes prolonged osteoblast differentiation and mineralization. This makes P2 a promising component for bone repair systems that are designed for challenging environments such as polytrauma. To address the inflammatory component of polytrauma, we incorporated MSCs into the hydrogel system. Their inclusion in the HA-P2 hydrogel is designed to attenuate systemic inflammation, creating a more favorable environment for bone regeneration.^22^ By combining MSCs with P2-loaded HA hydrogels, we aim to create a multifunctional therapeutic hydrogel capable of addressing both the inflammatory and osteogenic challenges of polytrauma.

This study investigated the osteo-immunomodulatory effects of HA hydrogels containing P2 and MSCs in a murine polytrauma model. We hypothesized that combining intrinsically disordered peptide P2 with MSCs in HA hydrogels would address both key deficits in polytrauma fracture healing: P2 would provide sustained osteoinduction through dynamic mineral-binding interactions that MSCs alone lack, while MSCs would modulate the hyperinflammatory environment that impairs bone regeneration, thereby achieving complete healing where either component alone is insufficient. The novelty of this study lies in addressing this critical therapeutic gap by combining MSCs with HA + P2. This represents the first application of these bioactive disordered peptides to augment MSC therapy specifically for polytrauma-impaired fracture healing. The study introduces two key innovations: First, we demonstrate that P2 provides sustained osteoinductive signaling that complements MSC immunomodulation, addressing both the inflammatory and osteogenic deficits characteristic of polytrauma. Second, we establish that this dual-action hydrogel achieves superior bone formation, mineralization, and trabecular organization compared to either peptide or MSC therapy alone in a severe osteotomy model. This work provides a clinically translatable solution for polytrauma patients with bone fractures, a population with significant unmet needs where current therapies fail to address the complex interplay between systemic inflammation and local bone regeneration.

## Materials and methods

### In vitro assays

#### Cell culture

Murine bone marrow-derived MSCs (AcceGen, Fairfield, NJ) were expanded on tissue culture plastic (TCP) in growth media (GM) composed of alpha minimum essential media (alpha-MEM) supplemented with 10% fetal bovine serum (GenClone, Genesee Scientific, San Diego, CA) and 1% penicillin–streptomycin (Gemini Bio Products, West Sacramento, CA) in standard cell culture conditions. GM was supplemented with 0.01 μM dexamethasone, 50 μg/mL L-ascorbic acid 2-phosphate, and 10 mM sodium β-glycerophosphate as osteogenic differentiation media (OM). GM or OM were refreshed every 48 h for the duration of the experiments. MSCs were maintained in culture until ~80% confluent and serially passaged until used at passage 5.^23^ MSCs (2.5 × 10^4^) were encapsulated in 80 µL hydrogels and cultured in 24 well plates.

#### Hydrogel synthesis

Pharmaceutical-grade hyaluronic acid (MW = 3 MDa) was acquired from H.T.L. SAS (Javené, France). The crosslinking agents 1,4-butanediol diglycidyl ether (BDDE) along with anhydrous sodium hydroxide and sodium chloride, were obtained from Merck KGaA (Germany). A 10 w/v% HA solution was prepared in 0.3 M NaOH under manual stirring. Subsequently, 1.6 v/v% BDDE and the mixture was maintained at 40°C for 4 h in a closed vessel. The resulting gel was placed in a cellulose membrane (MW cutoff = 14 kDa) and dialyzed against water (B.Braun, Melsungen AG, Melsungen, Germany) for 18 h. The gel was granulated by passage through a 130 µm mesh following dialysis. Water for injection and 10 wt.% sodium hyaluronate were added until a final hyaluronic acid concentration of 22 mg/mL was achieved. All steps were carried out in accordance with Good Laboratory Practice (GLP) within an ISO 13485-2016 certified facility as previously described.^24^

#### Peptide release from hydrogels

P2 was manufactured in accordance with ISO 13485:2016 and at peptide concentration as described in former studies.^25–29^ The peptide sequences of P2 are shown in Table 1.

**Table 1.** Peptide sequences of the two peptide variants.^30^

Peptide	Peptide sequence
P2	PLV PSQ PLV PSQ PLV PSQ PQ PPLPP

P: Proline; L: Leucine; V: Valine; S: Serine; Q: Glutamine; M: Methionine; H: Histidine – Covered by the patent.

The release of P2 peptide from the HA-based hydrogel was evaluated under physiological conditions. After the time point, the remaining distilled water in the well was extracted, the swollen hydrogel in the insert was weighted, and a Micro BCA™ Protein Assay Kit (Thermo Scientific, US) and a BioTek ELx800 Absorbance Microplate Reader (BioTek Instruments, Inc., Winooski, VT, USA) were used to measure the absorbance at 562 nm compared to a reference curve of 0.5, 1, 2.5, 5, 10, 20, 40, 200 µg/mL of albumin standard in distilled water. The reference curve was used to convert the absorbance to protein concentration. Hydrogel samples (*n* = 3) containing a known amount of P2 were incubated in phosphate-buffered saline (PBS, pH 7.4) at 37 °C with gentle agitation. At predetermined time points (0, 12, and 24 h), aliquots of the supernatant were collected and replaced with an equal volume of fresh PBS. We added 0.1 mL of HA + P2 (500 µg/mL, instead of 50 µg/mL) to 24-well inserts (0.4 µm, PET), then added 1 mL of distilled water to each well and incubated for 12 or 24 h.

The cumulative release of the P2 peptide (0–24 h) from the HA hydrogel was analyzed using the Korsmeyer–Peppas model^31^:

MtM∞=ktn

where MtM∞ is the fractional release at time *t*, *k* is the release constant, and *n* is the diffusional exponent indicative of the release mechanism. The logarithm of fractional release was plotted against the logarithm of time, and the parameters were obtained by linear regression. Only data within the early-time regime ( MtM∞ <0.6) were used for fitting. The best-fit parameters were *n* = 0.96 and *k* = 2.31 × 10^−2^ h^−n^ (*R*^2^ > 0.98), consistent with near case-II (relaxation-assisted) diffusion behavior.

#### Viability assay and DNA concentration

Cell viability was analyzed by a live/dead assay (LIVE/DEAD Viability/Cytotoxicity Kit) per the manufacturer’s protocol (Thermo Fisher, Waltham, MA). Fluorescent images were taken using confocal microscopy (Leica TCS SP8, Wetzlar, Germany). The percentage of living cells and cell shape descriptors were quantified through image analysis in Fiji ImageJ (version 1.51r; NIH, Maryland, USA).^32^ The total DNA content was quantified via Quant-iT™ PicoGreen assay (ThermoFisher) kit. Samples were scraped and collected in 300 µL of passive lysis buffer and sonicated with a probe sonicator (VCX 130PB, Sonics Vibra Cell) at 40% amplitude and 130 W power on ice three times for 10 s each for full homogenization of each sample. Samples (diluted at a 25:75 sample:1× TE buffer ratio) and standards (1000, 100, 10, 1, and 0 ng/mL) were diluted with 1× TE buffer. Samples were centrifuged at 5000*g* and 4° for 5 min and 100 µL of sample supernatant or standard were plated onto a black plate. Hundred microliters of 1× picogreen solution (diluted from 200× with 1× TE buffer) was added to each well and incubated for 5 min at room temperature. Fluorescence was read at 528/485 nm on a Synergy HTX plate reader. Standard curve was plotted and used to determine the DNA per sample accounting for the dilution of sample.^23^

#### Determination of osteogenic differentiation and cytokine profiling

Intracellular alkaline phosphatase (ALP) activity was determined by reducing p-nitrophenyl phosphatase (Sigma-Aldrich, St. Louis, MO).^33^ On Day 10 and 21, MSCs and hydrogels were fully dissolved in 1 M HCl for 48 h at 60°C, and calcium deposition was quantified using a Calcium Liquid Reagent Diagnostics Kit (Stanbio Labs, Boerne, TX).^33,34^ To determine secreted cytokines, MSCs were initially seeded in GM for 24 h to allow cell attachment. The media was then changed to GM containing 10% mouse serum (collected from C57BL/6J polytrauma mice at 72 h post-injury) for 48 h to simulate polytrauma conditions and precondition cells before exposure to osteogenic environments. This preconditioning protocol was applied to the following groups: (1) Monolayer MSCs (Polytrauma), (2) Hydrogel (HA), and (3) Hydrogel + P2 (HA + P2). Following the 48 h preconditioning period, the media was changed to OM, which was maintained for the duration of the experiment to evaluate the ability of our hydrogels to attenuate the inflammatory response under polytrauma-mimicking conditions while promoting osteogenic differentiation. Conditioned media was collected at 3 and 7 days after the transition to OM (corresponding to days 6 and 10 of total culture time) according to the manufacturer’s protocol, and the secreted protein levels of cytokines and growth factors were quantified by mouse cytokine antibody array analysis (C series 1000.1; RayBiotech, Inc., Norcross, GA, USA).

#### Animal surgery

We performed animal surgeries in the Department of Orthopaedic Surgery, University of California, Davis in compliance with the ARRIVE guidelines under an approved UC Davis Institutional Animal Care and Use Committee (IACUC) protocol (#23193). We acquired 60 10-week-old male C57BL/6J mice (Jackson Laboratories). Mice were randomly housed in groups of four and were acclimated to the housing vivarium for 2 weeks prior to any procedures. A water and pellet diet were provided ad libitum. At 12 weeks of age, animals were randomly divided into three treatment groups: (1) HA, (2) HA + P2, and (3) HA + P2 + MSCs, with 20 animals per group. Within each treatment group, animals were further allocated for specific downstream analyses: eight animals per group for calcified histology, µCT, and SAXS/XRD analysis (total 24 mice); eight animals per group for decalcified histology and immunohistochemistry (total 24 mice); and four animals per group for RNA sequencing (total 12 mice). Serum for cytokine analysis was collected from all animals at the time of euthanasia, allowing for larger sample sizes (*N* = 10 per group) for this blood-based assay.

Mice were injected subcutaneously with buprenorphine (0.1 mg/kg) for pain control and 100 µL saline for fluid support 5–10 min before surgery. For creating the femur fracture osteotomy, mice were anesthetized with 2%–4% isoflurane in oxygen and the right hind-limb was shaved and prepped in a standard sterile manner. An incision was made over the anterior knee joint, and the distal femur was exposed. The knee was flexed, and a 24G stainless steel needle was inserted through the femoral condyles as an intramedullary pin (IM) to provide fracture stabilization, following established protocols for murine femoral fracture models.^35,36^ A 4 mm mid-diaphyseal defect was then created using a 0.4 mm Gigli saw. The joint capsule was closed using Monocryl (Johnson & Johnson, Brunswick, NJ) sutures and the skin was closed with nylon sutures. X-ray imaging confirmed the correct placement of the needle and the femoral fracture osteotomy (Supplemental Figure 1A). The hydrogels (50–100 µL, approximately 3.2 µL/kg body weight) were then injected into the defect site using a 1 mL syringe with a blunt-tip needle, ensuring complete filling of the 4 mm osteotomy gap. The injectable nature of the hydrogel allowed for minimally invasive delivery directly into the fracture site while avoiding additional surgical trauma.

After fracture and while still under anesthesia, we induced blunt thoracic trauma by dropping a hollow aluminum cylindrical weight (~30 g) from a height of 55 cm through a vertical stainless-steel tube onto a Lexan platform resting on the animal’s chest (Supplemental Figure 1A). The impact was centered on the chest to ensure bilateral lung contusion and a consistent systemic inflammatory response while minimizing variability in injury severity. This apparatus has been described and characterized elsewhere,^37^ and our published results demonstrated successful polytrauma induction in mice using this method (Supplemental Figure 1B).^14^ Mice were allowed to recover from anesthesia in a well-ventilated area and kept warm using a heating pad underneath half of the cage with a surgical towel to minimize the risk of contact thermal injury. Mice were euthanized by exsanguination *via* cardiac puncture under anesthesia, followed by cervical dislocation. Blood was collected during cardiac puncture to analyze cytokines within the serum. Femur tissues were dissected for downstream analyses.

#### Analysis of cytokines within serum

Whole blood collected from cardiac puncture was processed for cytokine analysis within serum. The sample preparation was performed according to the company protocol (Cat#740446, Biolegend, San Diego, CA) and assessed on a BD Fortessa flow cytometer at the Institute of Regenerative Medicine, University of California, Davis. Cytokine concentrations were determined using BioLegend’s LEGENDplex data analysis software (Biolegend, San Diego, CA), and statistical comparisons between groups were performed using Prism 9.5.1 as described in the Statistical Analysis section.

#### 3D micro-computed tomography (µCT) analysis

We imaged femurs (fixed in 4% paraformaldehyde) using a microCT specimen scanner (Bruker microCT2242, Kontich, Belgium). Scan parameters were 55 kVp, 145 μA, 300 ms exposure time, an average of three exposures per projection, 0.5 mm aluminum filter, 500 projections per 180° and a 10 μm nominal voxel size. The raw images were calibrated using a hydroxyapatite (HAp) phantom of varying HAp concentrations. Noise in the images was reduced using a low-pass Gaussian filter with a sigma of 1.0 and a support of 2.0 release During post-processing, mineralized tissue was segmented using gray value thresholds of 71 (minimum) and 255 (maximum). Despeckle and sweep-type operations were utilized to remove scanning artifacts from the periphery of the sample region of interest. A region of interest (ROI) was contoured to include the 4 mm defect site plus 1 mm extensions on each end (total 6 mm ROI) to account for potential bone outgrowth and variability between samples due to anatomical differences between mice. Bone volume fraction (BV/TV) was determined by dividing the number of voxels denser than the low threshold representing mineralized tissue (BV: bone volume) by the total number of pixels in the region (TV: total volume). Several other parameters, including bone surface density (BS/TV), intersection surface (i.S), trabecular thickness (Tb.Th), trabecular separation (Tb.Sp), fractal dimension, and degree of anisotropy, were also measured.^38^

#### Histological, enzyme histochemical, and immunofluorescence analyses

After µCT imaging, we performed both calcified and decalcified histology. For decalcified samples, femurs were decalcified in 14% ethylenediaminetetraacetic acid (EDTA) solution with regular solution changes (every 3 days) until complete decalcification was achieved. We used Movat Pentachrome and Von Kossa/Van Gieson stains to evaluate the bone mineralization/non-mineralization balance over time. Movat Pentachrome stain was used to image different constituents of the connective tissue: mineralized bone appears bright yellow, cartilage appears as blue-green, and non-mineralized bone appears bright red.^39,40^ In Von Kossa/Van Gieson, mineralized bone is stained black versus non-mineralized bone in pink/red color.

Multiplex immunofluorescence was performed on 5 µm paraffin-embedded tissue sections using Opal fluorophores (Akoya Biosciences, Marlborough, MA) with a sequential staining approach adapted from our previous work^14^ and following Akoya Biosciences guidelines. Briefly, slides were baked at 60°C for 1 h, deparaffinized through a xylene and graded ethanol series, and endogenous peroxidase activity was quenched with 3% H₂O₂ for 15 min. Sections were blocked with 10% BSA and 0.05% Tween-20 in PBS for 30 min at room temperature. Primary antibodies were applied sequentially overnight at 4°C: CD31/PECAM-1 (1:500, AF3628, goat polyclonal, Novus Biologicals), Osterix (1:500, sc-393325 AC, mouse monoclonal, Santa Cruz), and Osteopontin (1:500, MAB808, mouse monoclonal, R&D Systems). Following each primary antibody incubation, sections were washed with TBST and incubated with species-appropriate HRP-conjugated secondary antibodies (Biocare Medical): goat-on-rodent polymer (GHP516) for CD31 (goat probe for 12 min, then goat polymer for 10 min) or mouse-on-mouse polymer for Osterix and Osteopontin (30 min). Signal amplification was achieved using Opal fluorophores diluted 1:100 in 1× amplification diluent: Opal 620 for CD31 (yellow), Opal 570 for Osterix (red), and Opal 650 for Osteopontin (cyan), each applied for 10 min. Between antibody cycles, slides underwent heat-mediated antigen retrieval (AR6 buffer) to strip bound antibodies while preserving the covalently bound fluorophore signal. After completion of all cycles, nuclei were counterstained with DAPI mounting medium (Invitrogen #2426204), coverslips were applied, and slides were dried overnight at room temperature in the dark before storage at 4°C. Sections were imaged using a Leica fluorescence microscopy system (Leica DM5500 photomicroscope equipped with a DFC7000 camera and operated by LASX software version 3.0, Leica Microsystem Ltd, Wetzlar, Germany).^41^

#### Quantitative histomorphometrical analysis

Sections were imaged at 20× magnification (3.09 pixel/μm) using a Leica microscopy system (Leica DM5500 photomicroscope equipped with a DFC7000 camera and operated by LASX software version 3.0, Leica Microsystem Ltd, Wetzlar, Germany). Histomorphometric analysis of stained sections was performed using Fiji ImageJ (version 1.51r; NIH, Maryland, USA) with the Trainable Weka Segmentation (TWS) plugin. The machine learning classifier was trained using one representative image per treatment group, randomly selected to avoid bias while ensuring all relevant tissue types and staining colors were present for accurate classification (mineralized bone, non-mineralized bone, cartilage, bone marrow, vasculature, and the fluorescence intensity of CD31, Osterix, and Osteopontin). The trained classifier was then applied to create an optimized script for analyzing tissue formation parameters including mineralization, new bone and cartilage formation, vascularization, and macrophage and neutrophil percentages across all samples. The histomorphometry measurements were performed as previously reported by Malhan et al.^42^

#### Small angle X-ray scattering/X-ray diffraction (SAXS/XRD) analysis

Technovit embedded bone sections (70 µm thickness) were used to study the collagen/hydroxyapatite (HAp) orientation and the size of HAp plates using SAXS/XRD analysis. Sections were measured at the synchrotron beamline P12,^43^ Petra III, Deutsches Elektronen-Synchrotron (DESY) Hamburg, operated by the European Molecular Biology Laboratory (EMBL).^44^ The beamline used an energy level of 18 keV with a sample-to-detector distance of 1.6 m and an asymmetric beam position concerning the detector center. At the beamline, a Pilatus 6 m detector was used. The accessible q-range ranged from 0.08 to 24 nm^−1^, which allowed us to measure the SAXS and WAXS signals in parallel. The 70 µm thick samples were mounted on a special holder, which could be mounted onto a piezo stage located in the sample exposure unit of the P12. This allowed us measuring the samples in a vacuum, significantly improving the scattering pattern quality.^43^ The slits restricted the beam size to 20 µm × 90 µm, which also determined the spatial resolution of this scan. Typically, a mesh scan of 1 mm × 2 mm was acquired. However, the bone growth on all samples was not always the same, and regions were adapted when needed to capture data in a representative area. Data reduction of the 2D scattering pattern to 1D scattering curves was performed using the BioSAXS software pipeline.^43^ Data analysis on the WAXS data, which yields parameters like crystal size and crystal structure, was performed using a Rietfieled analysis based on in-house developed Matlab scripts, MathWorks Inc., version 9.13.12. The SAXS signal was analyzed by our own developed Matlab scripts, MathWorks Inc., version 9.13.12 script as well. We extracted information on the orientation and degree of orientation of the HAp platelets as well as their thickness, the so-called T-parameter. To assess the mean crystal thickness, we applied the stack and card model developed by Gourrier et al.,^45^ adjusting it to the data. The T parameter serves as a measurement of HAp platelet size and indicates the thickness of the HAp platelets.^46^ The visualization of the different extracted parameters as 2D images and their analysis and correlation to the visual images obtained by microscopy allowed us to consider only areas with significant signal contribution within the bone samples, thus increasing the signal-to-noise ratio and decreasing fluctuations.

#### RNA isolation for RNA bulk sequencing analysis

Three experimental groups were analyzed, with four biological replicates per group: HA, HA + P2, and HA + P2 + MSCs. Bone samples were harvested under isoflurane anesthesia using a sterile technique and RNAzapped tools. After harvesting, samples were kept in RNAlater to prevent RNA degradation before the isolation. Using a homogenizer, the samples were lysed in TRIzol. RNA was isolated from bone samples using an RNA isolation kit from Qiagen and according to the company’s protocol (Qiagen, Hilden, Germany, Cat#NC9360823, Supplemental Figure 2). The purity of RNAs was examined using Nanodrop and Bioanalyzer machines. 3′-TaqSeq RNA Sequencing was performed on purified samples at the DNA Technologies Core, UC Davis.

#### Bulk sequencing data analysis

Isolated mRNA was reverse transcribed into complementary DNA (cDNA), fragmented, and ligated with Illumina sequencing adapters. Libraries were quality-checked and size-selected to ensure uniformity. Sequencing was performed at the UC Davis Genomics Core facility using the Illumina platform in single-end mode, generating high-quality reads for subsequent analysis. Raw sequencing reads were trimmed to remove low-quality bases and adapter sequences using Trimmomatic before being aligned to the mouse reference genome (GRCm39/mm39) using the STAR aligner (v2.7). Transcript assembly was performed using reference-guided methods with Cufflinks (v2.2.1), ensuring accurate mapping of reads to annotated exons and predicted exon junctions. Bioinformatics analysis of RNA-seq data was performed using the cloud analysis platform RaNAseq.^47^ The end-to-end pipeline consists of sequence processing steps, such as preprocessing of raw sequences and gene expression quantification using RSEM. Raw count data were normalized using the DESeq2 normalization method, which accounts for library size and RNA composition bias. Differential expression analysis was performed using the DESeq2 algorithm within RaNAseq. Genes with an adjusted *p*-value (false discovery rate, FDR) < 0.05 were considered significantly differentially expressed. No fold change cutoff was applied; all genes meeting the adjusted *p*-value threshold were included in the analysis. Pathway over-representation analysis and gene set enrichment analyses were performed utilizing Gene Ontology terms and KEGG pathways. Volcano plots and heatmaps were generated using the integrated visualization tools within RaNAseq.

#### Statistical analysis

Statistical analysis was performed using Prism 9.5.1 (GraphPad, San Diego, CA) for all analyses except SAXS/XRD data. Datasets were assessed for normality using the Kolmogorov-Smirnov test. Normally distributed data were summarized using mean and standard deviation, while non-normally distributed data were summarized using median and interquartile range. For normally distributed data, one-way or two-way ANOVA was performed followed by appropriate post-hoc tests. For non-normally distributed data, the Kruskal-Wallis test (non-parametric alternative to one-way ANOVA) was performed followed by Dunn’s multiple comparison test. Statistical analysis of SAXS/XRD data was performed using ANOVA in MATLAB (MathWorks Inc., version 9.13.12). Significant differences were presented as *p < 0.05, **p < 0.01, ***p < 0.001, and ****p < 0.0001.

## Results

### P2 peptide is released in a sustained manner from HA hydrogel

The P2 peptide exhibited sustained, progressive release from the HA hydrogel into the surrounding medium. After 12 h, 24.9 ± 2.6% of the peptide had been released, and an additional 23.5 ± 2.8% was released by 24 h, resulting in a cumulative release of 48.4%. The release profile showed a near-linear trend over the first 24 h, with no evidence of an initial burst phase. Fitting the release data to the Korsmeyer–Peppas kinetic model^48^ yielded parameters of *n* = 0.96 and *k* = 2.31 × 10^−2^ h^−n^ (*R*^2^ > 0.98), consistent with a near case-II (relaxation-assisted) diffusion mechanism. This indicates that peptide release was primarily governed by polymer relaxation and hydrogel swelling rather than simple Fickian diffusion, most likely due to weak electrostatic and hydrogen-bond interactions, resulting in this sustained, diffusion- and swelling-controlled release rather than a rapid burst. Correspondingly, the hydrogels swelled 249% after 12 h and 342% after 24 h, supporting the correlation between matrix expansion and controlled peptide release behavior (Supplemental Figure 3).^48,49^

### The hydrogels are biocompatible and have osteogenic capacity

**Figure 1.**
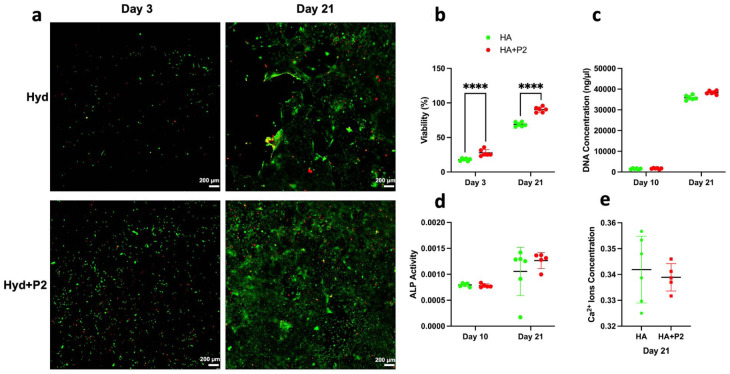
Intrinsically disordered peptide 2 and hydrogels are biocompatible with mesenchymal stem cells (MSCs) and induce osteogenic differentiation in these cells *in vitro*. (a) Representative images of the viability assay with green fluorescent calcein-AM to indicate intracellular esterase activity and red-fluorescent ethidium homodimer-1 to indicate the loss of plasma membrane integrity, Day 21 image is zoomed in to depict the differences in cell networks. (b) Quantitative analysis of cell viability in the two groups of hyaluronic acid-based hydrogels (HA) versus HA + P2 after 3 and 21 days of culturing MSCs in osteogenic media. (c) Quantitative analysis of DNA concentration in the two groups of hyaluronic acid-based hydrogels (HA) versus HA + P2 after 10 and 21 days of culturing MSCs in osteogenic media. (d and e) Quantifying the osteogenic differentiation of cultured MSCs with HA or HA + P2 using alkaline phosphatase (ALP), after 10 and 21 days and Calcium ions assays after 21 days. Data are mean ± standard deviation. *N* = 8.

Figure 1 illustrates the biocompatibility and osteogenic differentiation potential of hydrogels (HA) versus hydrogels containing P2 (HA + P2) when cultured with MSCs exposed to 10% polytrauma serum under osteogenic conditions. Qualitative assessment of cell viability was high at 3 and 21 days in both groups (Figure 1(a)), which was supported by quantitative analysis (Figure 1(b)) revealing that HA + P2 maintained a significantly higher percentage of viable cells than HA. We observed increased cellularity, indicated by DNA quantification (Figure 1(c)), for cells exposed to HA + P2. Compared to HA, we observed more uniform ALP activity at both 10 and 21 days, as well as increased calcium deposition by day 21 for HA + P2 constructs compared to HA alone, which had higher variability in response to polytrauma. Collectively, these findings underscore the superior osteogenic and biocompatibility properties of HA + P2, highlighting its potential utility in bone regeneration applications.

### Hydrogels containing P2 peptide reduce inflammation and enhance vascularization in mesenchymal stem cells pre-exposed to polytrauma serum

**Figure 2.**
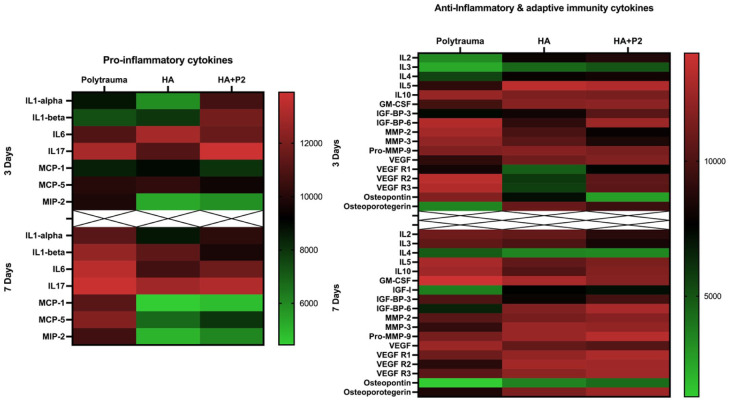
Intrinsically disordered peptide 2 and hydrogels induce anti-inflammatory effects on mesenchymal stem cells (MSCs) *in vitro*. A heat map of the secretome protein levels of inflammatory and angiogenesis cytokines. *N* = 8 samples/time point/group were pooled together for secretome analysis.

Figure 2 highlights the impact of hydrogels containing P2 on the secretome profile of MSCs exposed to polytrauma serum. The heat map demonstrates distinct changes in the secretion of inflammatory and angiogenesis-related cytokines across three experimental groups: MSCs alone exposed to polytrauma serum (Polytrauma), MSCs cultured with hydrogel (HA), and MSCs cultured with hydrogel containing P2 (HA + P2).

Key pro-inflammatory cytokines, including interleukin-6 (IL-6) as well as chemokines such as monocyte chemoattractant protein-1 (MCP-1), MCP-2, MCP-5, and macrophage inflammatory protein-1 alpha (MIP-2) were downregulated in the HA + P2 group compared to the polytrauma and HA groups 3 and 7 days of culture. In addition, IL-1 alpha and beta started downregulating in the HA + P2 group after 7 days of culture, suggesting reduced recruitment of inflammatory cells to the injury site. In contrast, angiogenesis-related factors such as vascular endothelial growth factor and its receptors (VEGF, VEGFR1, VEGFR2, and VEGFR3) as well as IL-10 and osteoprotegerin were upregulated in the HA + P2 group compared to both the Polytrauma and HA groups. This indicated that the inclusion of P2 within the hydrogel attenuated inflammatory responses and enhanced pro-angiogenic and anti-inflammatory signaling, leading to improved vascularization in the injury microenvironment. Overall, these findings suggest that HA + P2 hydrogels effectively modulated the secretome profile of MSCs, reducing inflammation while promoting angiogenesis.

### Hydrogels containing P2 or P2 peptide and mesenchymal stem cells reduce inflammation in polytrauma mice after 3 weeks of healing

**Figure 3.**
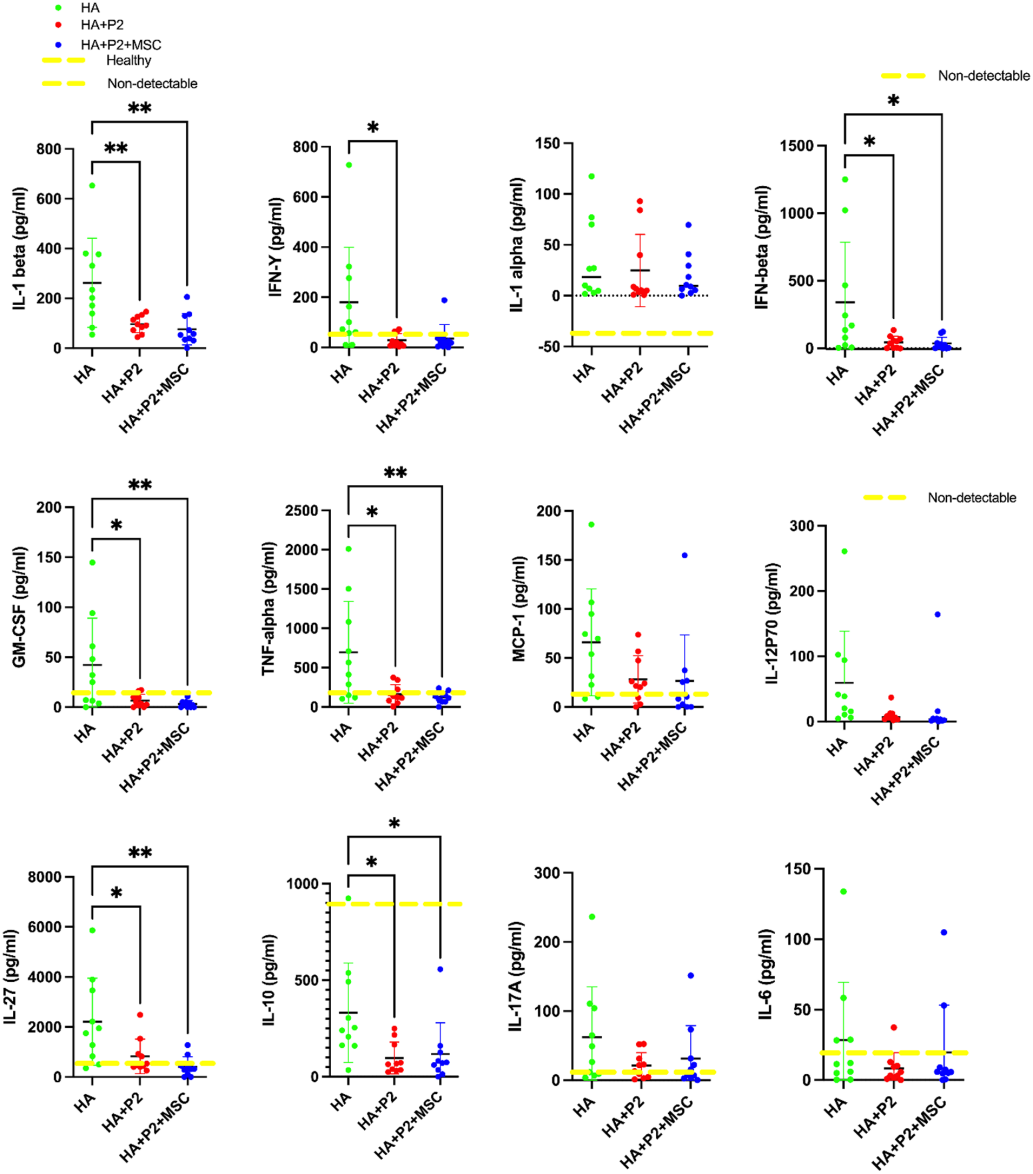
Intrinsically disordered peptide 2 and mesenchymal stem cells (MSCs) attenuate inflammatory response in polytrauma. The concentration of serum cytokines normalized to the healthy group (yellow line). *N* = 10. Significance is denoted as *p < 0.05, **p < 0.01. Data are mean ± standard deviation. *N* = 10.

After 3 weeks of healing, the inflammatory response in treated polytrauma mice was examined using a 13-plex inflammatory kit. Serum cytokine analysis revealed significant differences in inflammatory marker levels among the three experimental groups: hydrogel alone (HA), hydrogel with P2 (HA + P2), and hydrogel with P2 and mesenchymal stem cells (HA + P2 + MSCs). The HA + P2 + MSCs group demonstrated the most pronounced reduction in pro-inflammatory cytokine levels, including IL1-beta, IFN-beta, GM-CSF, tumor necrosis factor-alpha (TNF-α), IL-6 (Figure 3), and IL-23 (Supplemental Figure 4), approaching values observed in the healthy control group (yellow line). In contrast, animals treated with HA alone exhibited persistently elevated cytokine levels, indicative of ongoing systemic inflammation. The HA + P2 group showed intermediate reductions in these markers. Still, it was less effective compared to the HA + P2 + MSCs group regarding IL-1 alpha and MCP-1, highlighting the role of MSCs in attenuating the inflammatory response. Additionally, the normalization of IL-23 levels in the HA + P2 + MSCs group (Supplemental Figure 4) underscores its potential to mitigate the chronic inflammation associated with polytrauma. This reduction is critical, as elevated IL-23 has been implicated in impaired fracture healing due to its role in sustaining pro-inflammatory immune responses.^50^ These data suggest that combining P2 and MSCs within hydrogels effectively dampens systemic inflammation, creating a more favorable environment for tissue repair and regeneration.

### Hydrogels containing P2 peptide or P2 and mesenchymal stem cells enhance mineralization in polytrauma mice after 3 weeks of healing

**Figure 4.**
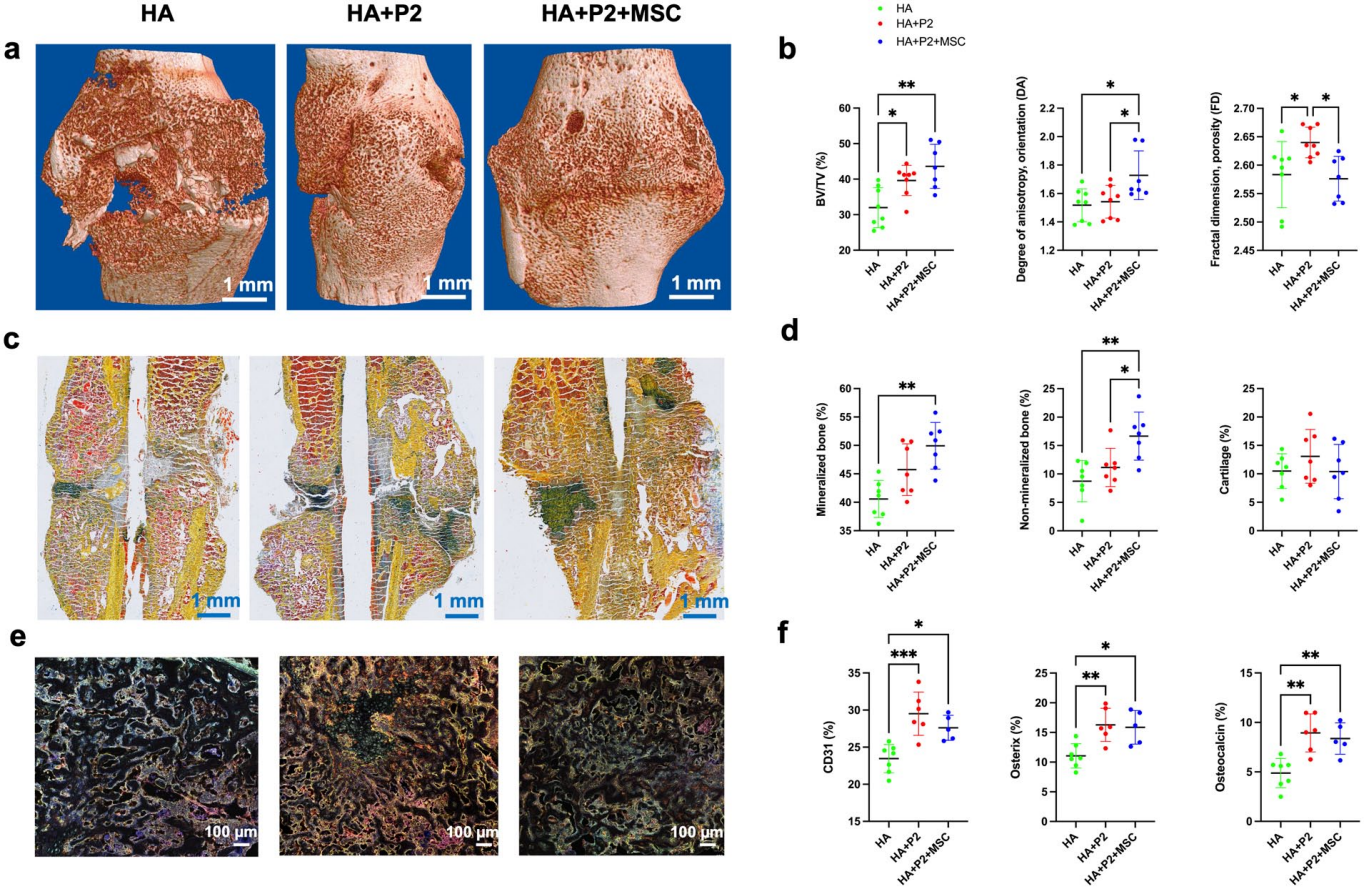
Intrinsically disordered peptide 2 and mesenchymal stem cells (MSCs) enhance fracture healing in polytrauma. (a) Representative reconstructed microCT (µCT) images of the femur in the three groups of hyaluronic acid-based hydrogels (HA), HA + P2, and HA + P2 + MSC after 3 weeks of healing. (b) Quantitative µCT analysis of bone volume fraction (BV/TV), degree of anisotropy (DA), for measuring orientation, and fractal dimension (FD). (c) Representative Movat Pentachrome histology staining for mineralized tissue (yellow, MB arrow), non-mineralized tissue (red, NMB arrow), cartilage formation (green, C arrow), bone marrow (brown, BM arrow; scale bars = 1 mm) after 3 weeks of healing. (d) Quantitative histological analysis of mineralized and non-mineralized tissue, as well as cartilage formation. *N* = 7 for a–d (scale bars = 1 mm). (e and f) Representative images and quantitative analysis of immunofluorescence analysis of CD31^+^ blood vessels (yellow color, BV arrow), Osterix (red, OX arrow), and Osteopontin (cyan, OP arrow) at the fracture site (images shown were taken from the center of the fracture site) after 3 weeks of healing (scale bars = 100 µm). In F, compared to our original power analysis (*N* = 8), the HA (*N* = 8), HA + P2 (*N* = 7), and HA + P2 + MSCs (*N* = 5) experienced discrepancies in sample size due to sample loss during preparation or attrition from the study. Data are mean ± standard deviation. Significant differences were presented as *p < 0.05, **p < 0.01, ***p < 0.001, and ****p < 0.0001.

The effects of the hydrogel treatments on bone formation and mineralization were assessed using µCT, histological staining, immunofluorescence, and quantitative histomorphometry. After 3 weeks of healing, the µCT analysis (Figure 4(a) and (b)) revealed significant differences in bone volume/tissue volume (BV/TV) percentage among the groups. HA + P2 and HA + P2 + MSCs groups exhibited significantly higher BV/TV than the HA group, indicating superior mineralized bone formation within the defect site after adding P2 and MSCs. Quantitative analysis (Figure 4(b) and Supplemental Figure 5) further highlighted significant improvements in other parameters of bone microarchitecture. The HA + P2 group exhibited significantly higher fractal dimension (FD), bone surface density (BS/TV), and intersection surface (i.S) compared to other groups. While higher FD and BS/TV typically indicate increased structural complexity and surface area, in the context of early fracture healing (21 days), these elevated values in the HA + P2 group likely reflect active callus formation and remodeling with increased trabecular branching and surface roughness rather than final mature bone architecture. In contrast, the HA + P2 + MSCs group demonstrated significantly higher degree of anisotropy (DA), indicative of improved tissue orientation and trabecular alignment, and trabecular thickness (Tb.Th), suggesting more advanced bone maturation. These complementary findings indicate that P2 promotes robust early callus formation with complex trabecular architecture, while the addition of MSCs enhances matrix organization and advances the bone toward a more mature structural state.

Histological analysis provided complementary insights into the mineralized and non-mineralized tissue composition. Representative Movat Pentachrome staining images (Figure 4(c)) revealed distinct tissue distributions within the defect site. Mineralized tissue (stained yellow) and non-mineralized tissue (stained red) were most prominent in the HA + P2 + MSCs group, indicating an active bone formation process after 21 days of healing. The HA + P2 group displayed an intermediate pattern with moderate mineralized tissue formation. Quantitative histological analysis (Figure 4(d)) confirmed these observations. Further validation of these findings was achieved using Von Kossa/Van Gieson staining (Supplemental Figure 6), which corroborated the Movat Pentachrome results. The HA + P2 + MSCs group exhibited extensive mineralized bone tissue (stained black), while the HA group showed sparse mineralization. These data collectively confirmed that the HA + P2 + MSCs treatment promoted robust and uniform bone mineralization, a key indicator of effective fracture healing. Additionally, immunofluorescence analysis (Figure 4(e) and (f)) revealed significant increases in the expression of key osteogenic markers, including osterix (OX) and Osteopontin (OP), in the HA + P2 and HA + P2 + MSCs groups compared to the HA group. These markers were concentrated at the center of the fracture site, indicating active osteogenesis. Furthermore, CD31^+^ blood vessels (yellow) were more abundant in the P2-containing groups compared to empty hydrogels, suggesting enhanced vascularization after adding P2, which is critical for supporting bone regeneration. In summary, the combined use of P2 and MSCs within hydrogels significantly enhanced bone formation and mineralization while improving tissue organization and vascularization in a polytrauma fracture model.

### Hydrogels containing P2 peptide or P2 and mesenchymal stem cells enhance bone maturity in polytrauma mice after 3 weeks of healing

The bone matrix is a composite structure made up of collagen and hydroxyapatite (HAp) platelets, with parameters such as size and orientation playing a critical role. HAp is mineralized between collagen fibers in mineralized tissue, and their growth is spatially restricted. As a result, platelets with a thickness of around a few nm and a length up to 20 nm develop with their c-axis aligned parallel to the collagen fibers. The exact structure depends on the stage of the remodeling. Thus, information extracted by SAXS/WAXS techniques on the platelet size, referred to as the T parameter,^45^ as well as the alignment of the HAp and collagen matrix, given their co-orientation, allows deducing of information on the remodeling and bone maturation. For this study, samples were sectioned along their longitudinal axis, and SAXS/XRD experiments were conducted to examine variations across the induced fracture site.

**Figure 5.**
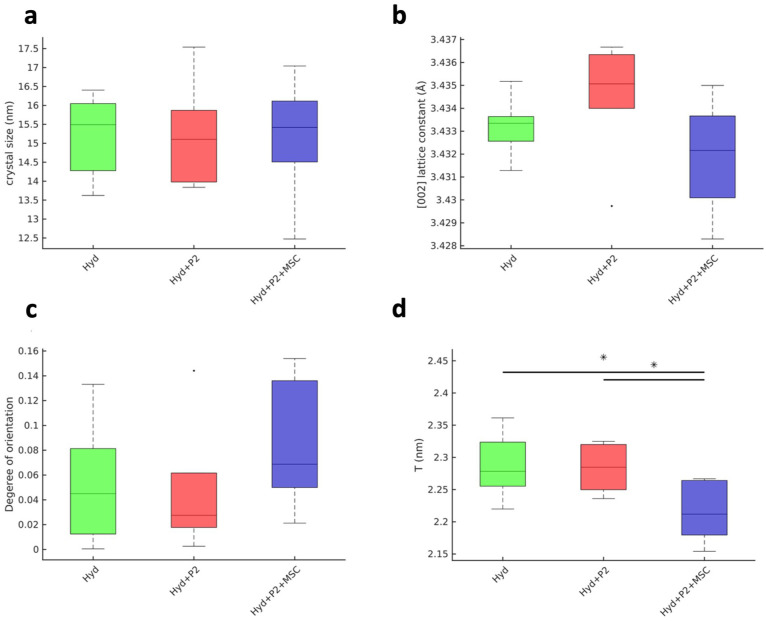
Intrinsically disordered peptide 2 (P2) and mesenchymal stem cells (MSCs) enhance bone maturity of fractured callus in polytrauma. Small angle X-ray scattering/X-ray diffraction (SAXS/XRD) analysis of the osteogenesis and biomineralization processes at the fracture site in the three groups of hyaluronic acid-based hydrogels (HA), HA + P2, and HA + P2 + MSC after 3 weeks of healing. (a) Quantitative analysis of the crystal size (a), lattice constant of the (002) reflection (b), degree of orientation (c), and *T* parameter (d). Significant differences were presented as *p < 0.05. The small black dots in the middle columns of b and c represent outliers. Data are mean ± standard deviation. *N* = 8.

Quantitative SAXS/XRD analysis (Figure 5(a)–(d)) demonstrated improvements in bone maturity metrics in the P2-containing hydrogel groups compared to the HA group. The crystal size (Figure 5A) and lattice constant of the (002) HAp reflection (Figure 5B), measures of crystallinity and structure of the HAp crystals, show no statistically significant difference between the studied samples, indicating that the mineralization process is most likely the same. The degree of orientation (Figure 5C) was highest in the HA + P2 + MSCs group, highlighting a more anisotropic and organized bone matrix with improved alignment of collagen and HAp platelets.

The *T*-parameter (Figure 5(d)), which represents the thickness of HAp platelets, showed differences among groups. In woven bone (immature bone), HAp platelets are typically larger but thinner and less organized. During bone maturation and remodeling, these platelets become thicker and more organized as the bone transitions toward lamellar bone. The HA + P2 group showed a *T*-parameter distribution around 2.5 nm after 8 weeks, which increased to 3.2 nm after 16 weeks, suggesting an intermediate stage of bone remodeling with ongoing maturation. The HA + P2 + MSCs group, exhibited both higher orientation and larger *T*-parameter values, together indicating a more advanced stage of bone formation and organization. The progression in *T*-parameter values, particularly when interpreted alongside degree of orientation, suggests active bone remodeling and maturation processes in the peptide-containing groups.

Supplemental Figures 7 and 8 further supported these findings by revealing differences in mineralized matrix organization among groups. Supplemental Figure 9 focused on the spatial distribution of mature bone within the callus, with the HA + P2 + MSCs group demonstrating more extensive distribution of mineralized regions throughout the defect site. The observed improvements in HAp organization and mineral density highlighted the potential of the HA + P2 + MSCs system for accelerating and improving bone healing in polytrauma cases.

### Hydrogels containing P2 peptide or P2 and mesenchymal stem cells enhance osteogenic and anti-inflammatory genes in polytrauma mice after 3 weeks of healing

To understand the molecular effects of different hydrogel formulations on fracture healing under polytrauma conditions, we performed bulk RNA sequencing of femoral defect tissues harvested at 3 weeks post-injury. Three groups were compared: HA, HA + P2, and HA + P2 + MSCs.

**Figure 6.**
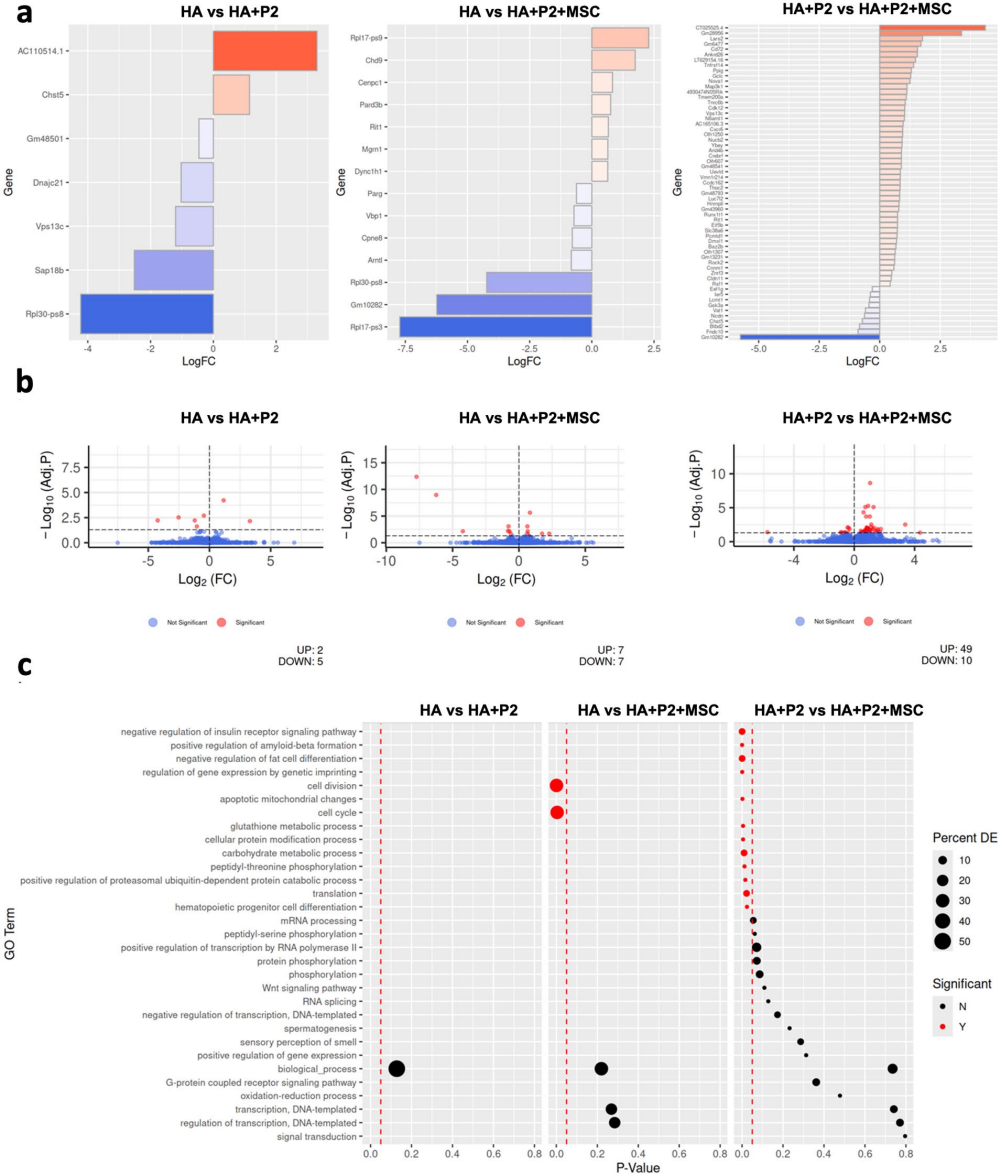
The combination of intrinsically disordered peptide 2 (P2) and mesenchymal stem cells (MSCs) both upregulates osteogenesis and downregulates inflammatory genes at the fracture site after 3 weeks of healing. (a) Log2 fold gene expression changes for all significant (adjusted *p* < 0.05) differentially expressed genes by comparison. Up-regulated genes were more highly expressed by the second group listed in the comparison. (b) Volcano plots detailing the differential gene expression analysis results for each comparison. Each point represents a single gene, corresponding to the log fold change and −log10 (adjusted *p*-value). Genes with a positive log fold change are more highly expressed by the second group listed in the comparison. (c) Results of gene ontology enrichment analysis for each comparison. Dots are positioned according to their *p*-value with significant *p*-values (<0.05) being displayed in red. Size of dot corresponds to the percent of genes labeled with the term that were significantly differentially expressed. Missing dots correspond to missing data from the tables provided. *N* = 4.

**Figure 7.**
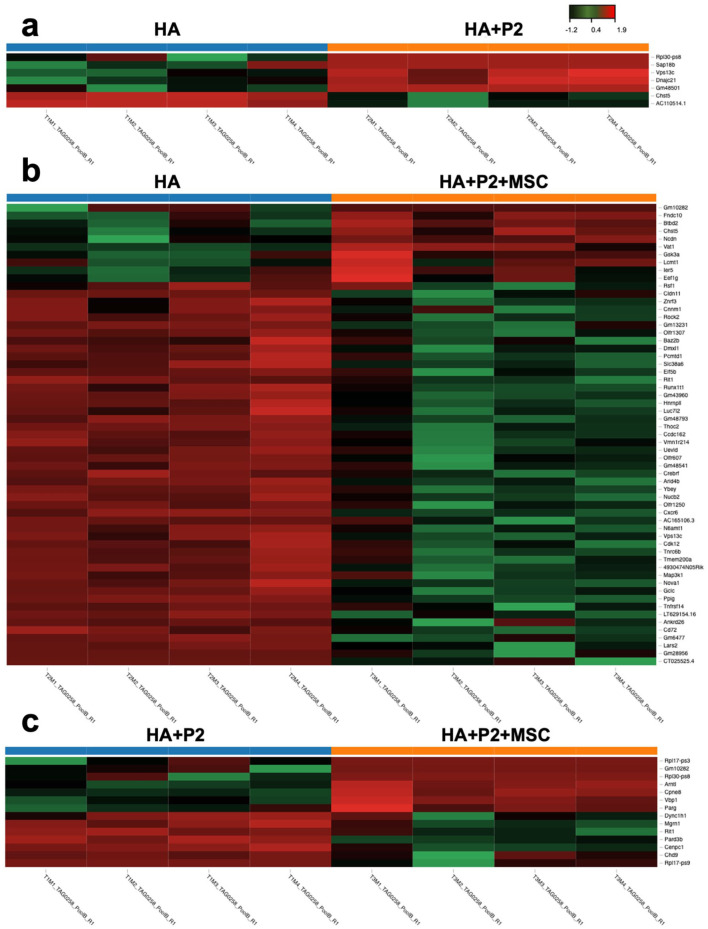
Differential gene expression among hydrogel treatment groups. Heatmaps illustrating gene expression changes among: (a) Hyaluronic acid-based hydrogel (HA) versus HA + P2: Upregulation of genes related to cartilage and bone healing, including extracellular matrix (ECM) organization and collagen biosynthesis, in the HA + P2 group. (b) HA versus HA + P2 + MSCs: Downregulation of inflammatory pathways (e.g. IL-2, IL-3, and IL-5 signaling) and upregulation of bone mineralization pathways in the HA + P2 + MSCs group. (c) HA + P2 versus HA + P2 + MSCs: Enhanced metabolic activity, including mitochondrial metabolism and phagosome pathways, and increased cell-cell interactions in the HA + P2 + MSCs group. Color scale indicates log₂ fold change in gene expression (red: upregulated; green: downregulated). *N* = 4.

This comparison revealed seven differentially expressed genes, five of which were upregulated in the HA + P2 group (Supplemental Figure 10 and Supplemental Table 1). GO analysis (Supplemental Table 2 and Figure 6(c)) suggested potential enrichment in pathways associated with matrix remodeling and early osteogenesis including glycosaminoglycan degradation, collagen biosynthesis, and mineral absorption. These changes support the pro-osteogenic effect of P2. The corresponding volcano plot (Figure 6(b)) and heatmap (Figure 7(a)) highlight these expression shifts.

Fourteen genes were significantly differentially expressed between HA and HA + P2 + MSCs (seven upregulated, seven downregulated; Supplemental Figure 10 and Supplemental Table 3). GO enrichment (Supplemental Table 4 and Figure 6(c)) identified pathways related to cell cycle control, E2F transcriptional regulation, and TP53-mediated DNA repair, alongside a downregulation of IL-2, IL-3, and IL-5 signaling pathways. These transcriptomic trends are consistent with a potential dual role for MSCs in promoting repair and modulating inflammatory pathways. Visual summaries are shown in Figure 6(a) and (b), as well as Figure 7(b).

This comparison yielded the most pronounced differences, with 59 differentially expressed genes, 49 upregulated and 10 downregulated in the HA + P2 + MSCs group (Supplemental Table 5 and Supplemental Figure 10). Enriched pathways (Supplemental Table 6) included fatty acid metabolism, phospholipid biosynthesis, GDP-mannose metabolism, and endocytosis/phagocytosis, suggesting enhanced cellular communication and metabolic activation. Notably, IL-3 signaling was the only inflammatory pathway significantly downregulated in this comparison, indicating a fine-tuned immunomodulatory role. These results are visualized in Figure 6(a) and (b) as well as Figure 7(c).

Together, these transcriptomic findings suggest that P2 can promote early osteogenic and matrix-related gene expression, while MSCs appear to modulate additional epigenetic, metabolic, and anti-inflammatory regulation. Their combination (HA + P2 + MSCs) showed additive expression patterns consistent with the enhanced healing outcomes observed across structural, cellular, and molecular assessments.

## Discussion

Our results indicate that combining P2 and MSCs within a hyaluronic acid hydrogel enhances bone formation, reduces systemic inflammation, and improves matrix organization in a polytrauma fracture model. These combined effects support the therapeutic potential of this hydrogel for complex bone injuries. While HA and MSCs have been previously investigated for bone repair, these systems often lack sustained bioactivity and tunability under inflammatory conditions. Covalently modified HAs, such as those conjugated with BMP-2–derived peptides (e.g. the knuckle epitope), have shown improved osteoinductivity but are limited by burst release, rapid clearance, or the need for complex delivery systems.^21^ In our recent study,^16^ we evaluated HA + MSCs in a murine polytrauma model with closed femoral fracture. That study demonstrated that while local MSC delivery via HA hydrogel improved outcomes compared to systemic MSC administration, it was insufficient to achieve complete fracture healing in the polytrauma setting. This finding highlighted the need for a dual-action approach that addresses both the osteogenic and immunomodulatory challenges in polytrauma. Therefore, in the current study, we incorporated the osteogenic peptide P2 to enhance the bone-forming capacity of the HA + MSC system. The intrinsically disordered structure of P2 enables sustained and dynamic interaction with the extracellular environment, offering continuous osteogenic signaling without covalent tethering or rapid degradation. The combination of P2-driven osteogenesis and MSC-mediated immunomodulation demonstrated enhanced outcomes, including improved mineralization, structural organization, and reduced inflammation. These results suggest that P2 provides critical osteoinductive signals that work in concert with MSC immunomodulation to create a more favorable regenerative environment, addressing the limitations observed in our previous work with HA + MSCs alone.

Beyond IDPs developed in our lab including P2, other IDPs have been extensively studied for their roles in bone mineralization. Osteopontin, a highly phosphorylated IDP abundant in bone, is rich in acidic residues and capable of binding calcium ions, thereby regulating hydroxyapatite crystal growth and inhibiting mineral over-aggregation.^51^ Its flexible, unstructured domains allow it to interact with both the mineral and organic phases of the matrix, making it essential for bone remodeling and repair. Similarly, dentin matrix protein 1 (DMP1) exists in multiple isoforms and undergoes proteolytic processing into N-terminal and C-terminal fragments, which distinctly regulate mineral nucleation and hydroxyapatite crystal growth.^52^ These naturally occurring IDPs not only control mineral deposition but also influence osteocyte differentiation and matrix organization. While Osteopontin and DMP1 are integral to physiological mineralization, P2 offers a synthetic alternative designed to mimic these functionalities with greater tunability. Its proline-rich sequence emulates the mineral-binding domains of endogenous IDPs, enabling sustained osteogenic activity in biomaterial systems, especially under challenging conditions such as polytrauma.

P2, an intrinsically disordered peptide, promoted osteogenesis, as evidenced by increased bone formation and mineralization in µCT and histology along with a higher degree of ultrastructure organization, as proven by the SAXS data. These findings align with our previous studies demonstrating P2’s ability to promote biomineralization and collagen alignment in bone tissue.^4,53^ MSCs further contributed to fracture healing by modulating the inflammatory environment, as reflected by the downregulation of inflammatory cytokine pathways, including IL-2, IL-3, and IL-5 signaling. Notably, the combination of P2 and MSCs produced superior outcomes, with increased bone mineralization, reduced systemic inflammation, and improved structural organization at the fracture site. These results suggest that the dual mechanism, P2-driven osteogenesis and MSC-mediated immunomodulation, creates a regenerative microenvironment conducive to accelerated and enhanced fracture healing.

An essential aspect of successful bone regeneration is the establishment of adequate vascularization to support tissue repair and remodeling.^54–56^ Our secretome analysis revealed increased expression of pro-angiogenic factors such as vascular endothelial growth factor (VEGF) and angiopoietin-1 (Ang-1) in the HA + P2 + MSCs group, highlighting the role of MSCs in promoting vascularization through paracrine signaling. These findings were corroborated in vivo by immunofluorescence data, demonstrating a significant increase in CD31^+^ endothelial cells in the fracture site of the HA + P2 and HA + P2 + MSCs groups compared to the hydrogels alone. This suggests enhanced vascular network formation, which is critical for nutrient delivery, waste removal, and osteogenic signaling during bone repair. The combined effects of P2 and MSCs modulated inflammation, promoted osteogenesis, and created a pro-angiogenic environment that further supported fracture healing.

Our murine polytrauma model combining femoral fracture with chest trauma successfully recapitulates clinical polytrauma pathophysiology.^14^ The model reproduces systemic inflammatory dysregulation, sustained innate immune activation with adaptive suppression, pulmonary injury, and impaired fracture healing. These findings align with Mangum et al.,^13^ who showed that polytrauma (burn + femur fracture + thoracic trauma) in rats caused substantial bone volume reduction and altered leukocyte kinetics, confirming that additional injuries create a “second hit” disrupting normal fracture healing.^13^ The substantial inter-animal variability we observed, while initially a statistical challenge, enhances clinical relevance. Mangum et al. reported similar heterogeneity in their polytrauma model, and McKinley et al.^57^ identified comparable variability in clinical polytrauma patients with similar injury severity.^57^ This biological heterogeneity represents a model strength, capturing complexity often masked in homogeneous experimental designs. The reproducible induction of key pathological features across multiple studies validates model utility, while outcome variability mirrors clinical reality. Future studies should account for this variability in sample size calculations and consider phenotype stratification to identify subgroup-specific therapeutic targets.

Although our polytrauma fracture model incorporated an osteotomy, a more severe injury than the blunt fracture model used in previous studies^14^ leading to dysregulated healing, the combination of P2 and MSCs significantly improved healing outcomes, suggesting their ability to counteract the deleterious effects of polytrauma locally. Additionally, we observed reduced variability in fracture healing in the HA + P2 and HA + P2 + MSCs groups *in vivo*. This further highlights the robustness of HA + P2 + MSCs in promoting consistent regenerative outcomes.

One of the most compelling aspects of the HA + P2 and HA + P2 + MSCs systems is their feasibility for clinical translation. The injectable and minimally invasive nature of these hydrogels makes them particularly suited to clinical settings where additional surgery may not be feasible. The ease of use and localized delivery offered by these hydrogels significantly reduce the risk of complications associated with surgical implantation and improve patient compliance, highlighting their potential for widespread clinical application.

In polytrauma scenarios, the body’s metabolic and epigenetic landscapes undergo significant alterations that can impede effective bone healing. In our study, transcriptomic trends suggest activation of pathways related to mitochondrial metabolism and phospholipid biosynthesis, which may support the increased energy demands of tissue repair and regeneration. Additionally, upregulation of genes associated with E2F-mediated transcription and TP53-regulated DNA repair could reflect adaptive cellular responses to injury. While these findings align with existing literature on trauma-induced reprogramming, further validation is required to confirm epigenetic regulation at functional level.

The therapeutic potential of HA + P2 and HA + P2 + MSCs extends to a range of clinical scenarios, particularly for polytrauma patients and those at high risk of nonunion fractures. This treatment’s ability to address both inflammation and bone regeneration could reduce recovery times and improve patient outcomes. Furthermore, the modular nature of the hydrogel system allows for tailored applications, such as localized delivery of P2 alone for early-stage fractures or the addition of MSCs for more severe injuries requiring enhanced immunomodulation.

While the findings are promising, several limitations must be addressed in future work. First, the absence of an HA + MSCs-only group in the current study limits our ability to definitively quantify the individual contribution of P2 versus the combined effect with MSCs within this specific experimental context. However, our recent study evaluating HA + MSCs in a polytrauma closed fracture model,^16^ demonstrated that while local MSC delivery improved outcomes compared to systemic delivery, it was insufficient to achieve complete fracture healing in polytrauma conditions. Specifically, HA + MSCs alone did not adequately restore bone formation or fully resolve the prolonged inflammatory response characteristic of polytrauma. This finding informed our decision to focus the current study on evaluating whether the addition of the osteogenic peptide P2 to the HA + MSCs system could enhance healing outcomes in an even more severe injury model (osteotomy versus closed fracture). The improved bone formation, enhanced mineralization, and reduced inflammation observed with HA + P2 + MSCs compared to HA + P2 alone in the present study suggests that MSCs and P2 provide complementary benefits-P2 driving osteogenesis while MSCs modulate inflammation. P2 has demonstrated consistent biochemical stability and biocompatibility across multiple independent in vitro assays^20,25,26,58–62^ and in prior in vivo studies.^16^

. In the present work, no adverse local or systemic tissue responses were observed, further confirming the safety of P2 and its degradation products within the HA matrix. Collectively, these findings support the suitability of P2 for controlled local delivery applications, and future studies will focus on detailed analyses of in vivo degradation kinetics and pharmacokinetics to substantiate its translational potential. Together, these studies demonstrate a progressive refinement of the therapeutic approach: from establishing that local MSC delivery is superior to systemic delivery, to demonstrating that combining MSCs with osteoinductive peptides further enhances regenerative outcomes in severe polytrauma scenarios. Future studies with all four groups (HA, HA + P2, HA + MSCs, and HA + P2 + MSCs) evaluated in parallel would be valuable to fully characterize the individual and combined contributions of each component within a single experimental design. Second, the study focused on a single time point (21 days), which restricts insights into the dynamic progression of healing. A longitudinal analysis would enable a more comprehensive understanding of how the treatment influences different stages of fracture repair. Third, the life-span and activity of the delivered MSCs was not tracked. Future studies should incorporate additional control groups, biomechanical testing, in vivo tracking of cell deliveries, and longer observation periods to address these gaps. To build on these findings, future studies should extend the evaluation period to 3 or 6 months to better understand the long-term effects of HA + P2 and HA + P2 + MSCs on fracture healing and bone remodeling. Additionally, studies in large animal models that mimic the biomechanical and physiological aspects of the human skeleton are necessary to validate the clinical translatability of these treatments.^63,64^ Finally, exploring cell-type-specific effects through single-cell RNA sequencing could provide deeper insights into the molecular mechanisms underlying the observed outcomes.

## Conclusions

This study highlights the potential of combining P2 and MSCs within a hydrogel to enhance bone regeneration in a polytrauma nonunion fracture osteotomy model. P2 significantly improved osteogenesis, while MSCs effectively modulated inflammation, and their combination demonstrated accelerated bone maturation and reduced variability in healing outcomes. Notably, the advanced bone formation observed at the early 21-day time point underscores the ability of this treatment to address both inflammatory and osteogenic challenges in severe injury scenarios. The injectable and user-friendly nature of these hydrogels offers a minimally invasive alternative to traditional surgical approaches, making them particularly suitable for polytrauma patients who may not tolerate extensive procedures. Furthermore, the modular design allows for tailored applications based on injury severity and patient needs. In summary, the HA + P2 and HA + P2 + MSCs systems represent a novel and effective approach for improving bone regeneration in challenging polytrauma fracture environments.

## Supplemental Material

sj-docx-1-tej-10.1177_20417314251397106 – Supplemental material for Mesenchymal stem cells delivered via a bioactive disordered peptide-hydrogel platform modulate early inflammation and enhance skeletal repair in a polytrauma model Supplemental material, sj-docx-1-tej-10.1177_20417314251397106 for Mesenchymal stem cells delivered via a bioactive disordered peptide-hydrogel platform modulate early inflammation and enhance skeletal repair in a polytrauma model by Augustine Mark Saiz, Maryam Rahmati, Tony Daniel Baldini, Aneesh Satish Bhat, Soren David Johnson, Mengyao Liu, Renato Miguel Reyes, Shierly W. Fok, Mark A. Lee, Thaqif El Khassawna, D. C. Florian Wieland, André Lopes Marinho, Clement Blanchet, J. Kent Leach and Håvard Jostein Haugen in Journal of Tissue Engineering
