# Supplementary material for: Mesenchymal stem cells delivered via a bioactive disordered peptide-hydrogel platform modulate early inflammation and enhance skeletal repair in a polytrauma model
Source: J Tissue Eng. 2025 Dec 1;16:20417314251397106. doi: 10.1177/20417314251397106 (PMC12669493; doi:10.1177/20417314251397106)
Supplement: sj-docx-1-tej-10.1177_20417314251397106 – Supplemental material for Mesenchymal stem cells delivered via a bioactive disordered peptide-hydrogel platform modulate early inflammation and enhance skeletal repair in a polytrauma model [file sj-docx-1-tej-10.1177_20417314251397106.docx]

**Mesenchymal Stem Cells Delivered via a Bioactive Disordered Peptide-Hydrogel Platform Modulate Early Inflammation and Enhance Skeletal Repair in a Polytrauma Model**

*Augustine Mark Saiz^1,2^*, Maryam Rahmati^1,2^*, Tony Daniel Baldini^1,3^, Aneesh Satish Bhat^1^, Soren David Johnson^1^, Mengyao Liu^1,3^, Renato Miguel Reyes^1^, Shierly W. Fok^1,4^, Mark A. Lee^1^, Thaqif El Khassawna^5,6^, D.C. Florian Wieland^7^, André Lopes Marinho^7^, Clement Blanchet^8^, J. Kent Leach^1,4^, Håvard Jostein Haugen^2#^*

*^1^Department of Orthopaedic Surgery, UC Davis Health, 4860 Y Street, Suite 3800, Sacramento, CA, 95817, USA*

*^2^Department of Biomaterials, Institute of Clinical Dentistry, University of Oslo, 0318 Oslo, Norway*

*^3^California Northstate University College of Medicine, 9700 W Taron Drive, Elk Grove, CA*

*95757, USA*

*^4^Department of Biomedical Engineering, UC Davis, Davis, CA 95616 USA*

*^5^Experimental Trauma Surgery, Justus-Liebig University Giessen, Giessen, Germany*

*^6^School of Pharmacy, The University of Jordan, Amman 11942, Jordan*

*^7^Institute of Metallic Biomaterials, Helmholtz Zentrum Hereon, Max-Planck-Straße 1, 21502 Geesthacht, Germany*

*^8^European Molecular Biology Laboratory EMBL, Hamburg Site, c/o DESY Notkestrasse 85, 22603, Hamburg, Germany*

** These authors contributed equally to this study.*

*#Email of the Corresponding Author:* [*h.j.haugen@odont.uio.no*](mailto:h.j.haugen@odont.uio.no)


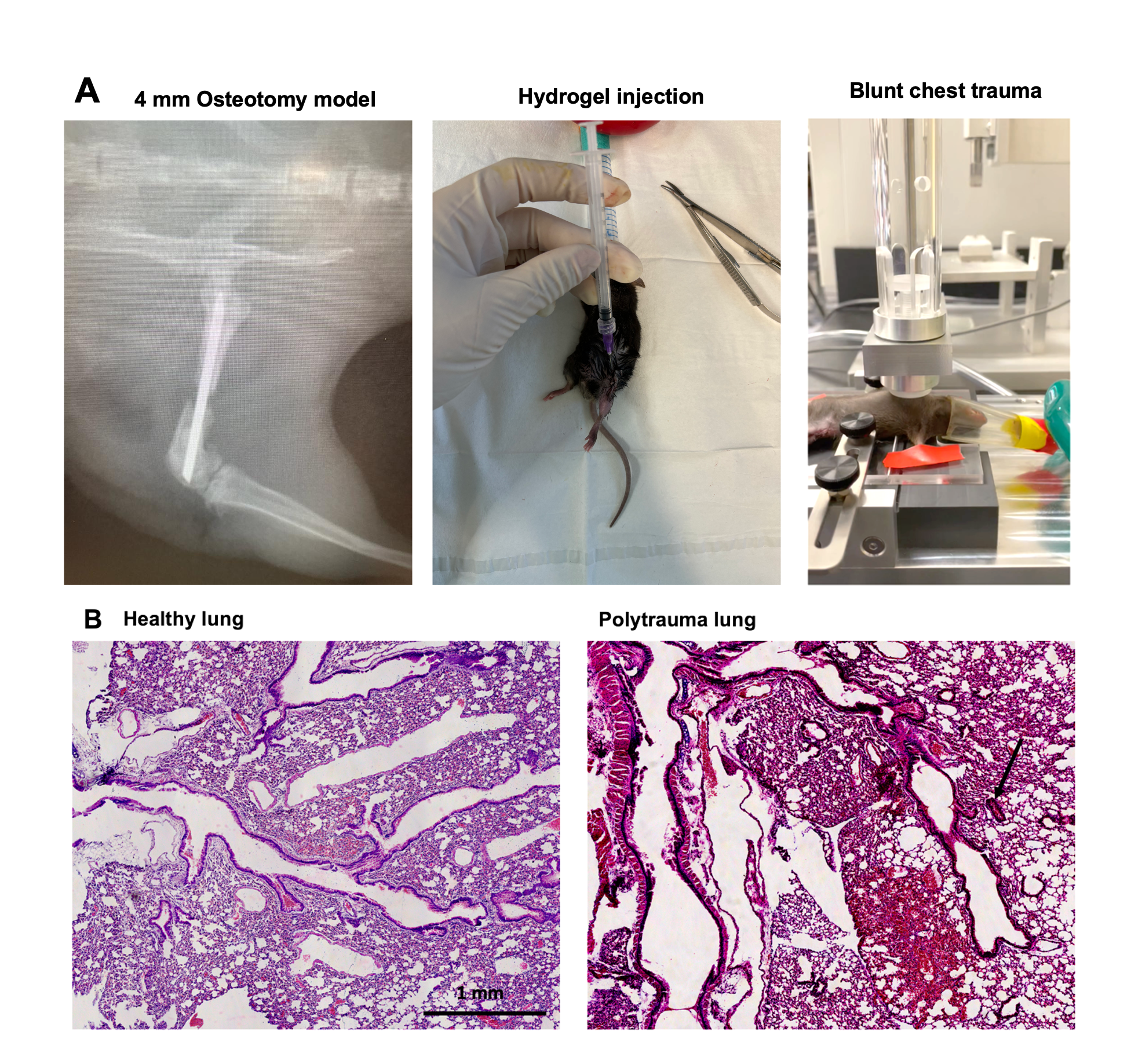


**Supp Figure 1.** Representative images of the polytrauma model (femur osteotomy, 4 mm defect size, + chest trauma). **(A)** Representative images from left to right of inducing polytrauma through inserting a 24 G needle as our intramedullary pin (IM), creating a 4 mm defect, radiographical assessment of the defect site, injecting our hydrogels and inducing the blunt fracture using an Einhorn drop weight device, and inducing the chest trauma using a standard drop weight device. **(B)** Representative images of the Hematoxylin & Eosin (H&E) stained lung tissues from healthy and polytrauma mice, with a black arrow indicating erythrocytes. Scale bar = 1 mm.

**
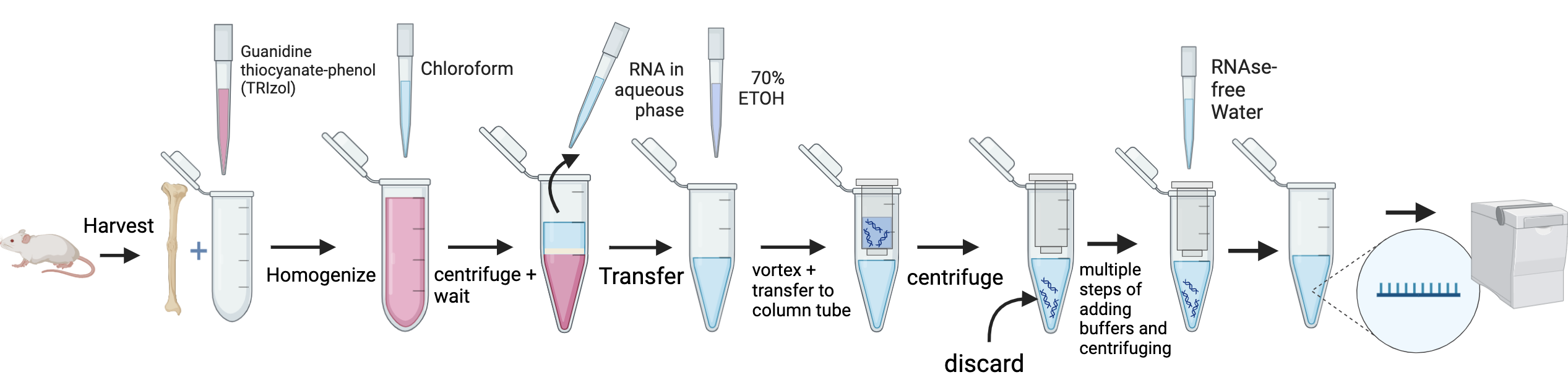
**

**Supp Figure 2.** A schematic of RNA isolation steps using a Qiagen kit from the fractured callus.

**
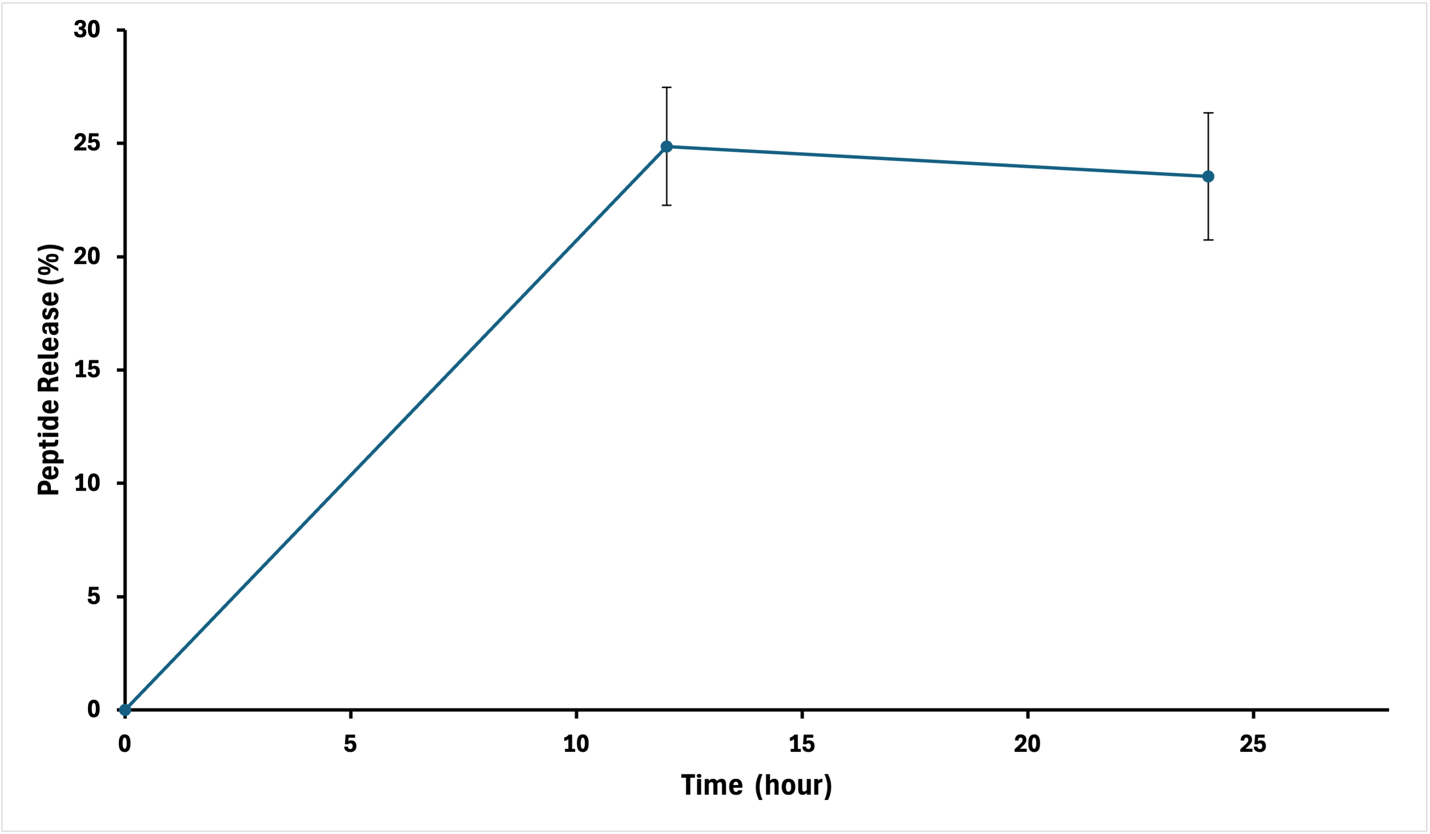
**

**Supp Figure 3*.*** *In vitro release profile of bioactive peptide P2 from HA hydrogel formulations. Cumulative release of peptide P2 from the HA-based hydrogel was measured over time under physiological conditions (37 °C in PBS). The release followed a sustained, near-linear trend up to 24 hours without an initial burst phase. Fitting to the Korsmeyer–Peppas model yielded a diffusion exponent n=0.96 and release* k=2.31×10^−2^ h^−n^ *(R² > 0.98), indicating a near case-II (swelling/relaxation-assisted) diffusion mechanism. Error bars represent standard deviation (N = 3).*

**Kinetic Modeling of Peptide Release**

The cumulative release of the P2 peptide (0–24 h) from the HA hydrogel was analyzed using the **Korsmeyer–Peppas model (1)**:

$$\frac{M_{t}}{M_{\infty}=kt^{n}}$$

where$\frac{M_{t}}{M_{\infty}}$is the fractional release at time *t, k* is the release constant, and n is the diffusional exponent indicative of the release mechanism. The logarithm of fractional release was plotted against the logarithm of time, and the parameters were obtained by linear regression. Only data within the early-time regime ($\frac{M_{t}}{M_{\infty}}$<0.6) were used for fitting. The best-fit parameters were n=0.96 and k=2.31×10^−2^ h^−n^, (R² > 0.98), consistent with near case-II (relaxation-assisted) diffusion behavior. This result suggests that peptide release was predominantly governed by hydrogel swelling and polymer relaxation rather than by pure Fickian diffusion.

**
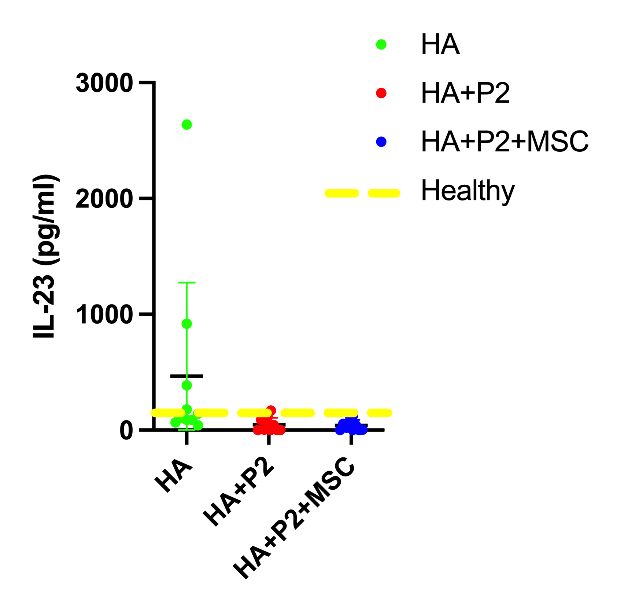
**

**Supp Figure 4.** Intrinsically disordered peptide 2 and mesenchymal stem cells (MSCs) attenuate inflammatory response in a polytrauma. The serum cytokine interleukin 23 (IL-23) concentration was normalized to the healthy group (yellow line). N=10. Significant differences between groups were presented. Data are mean +/- standard deviation. N=10.

**
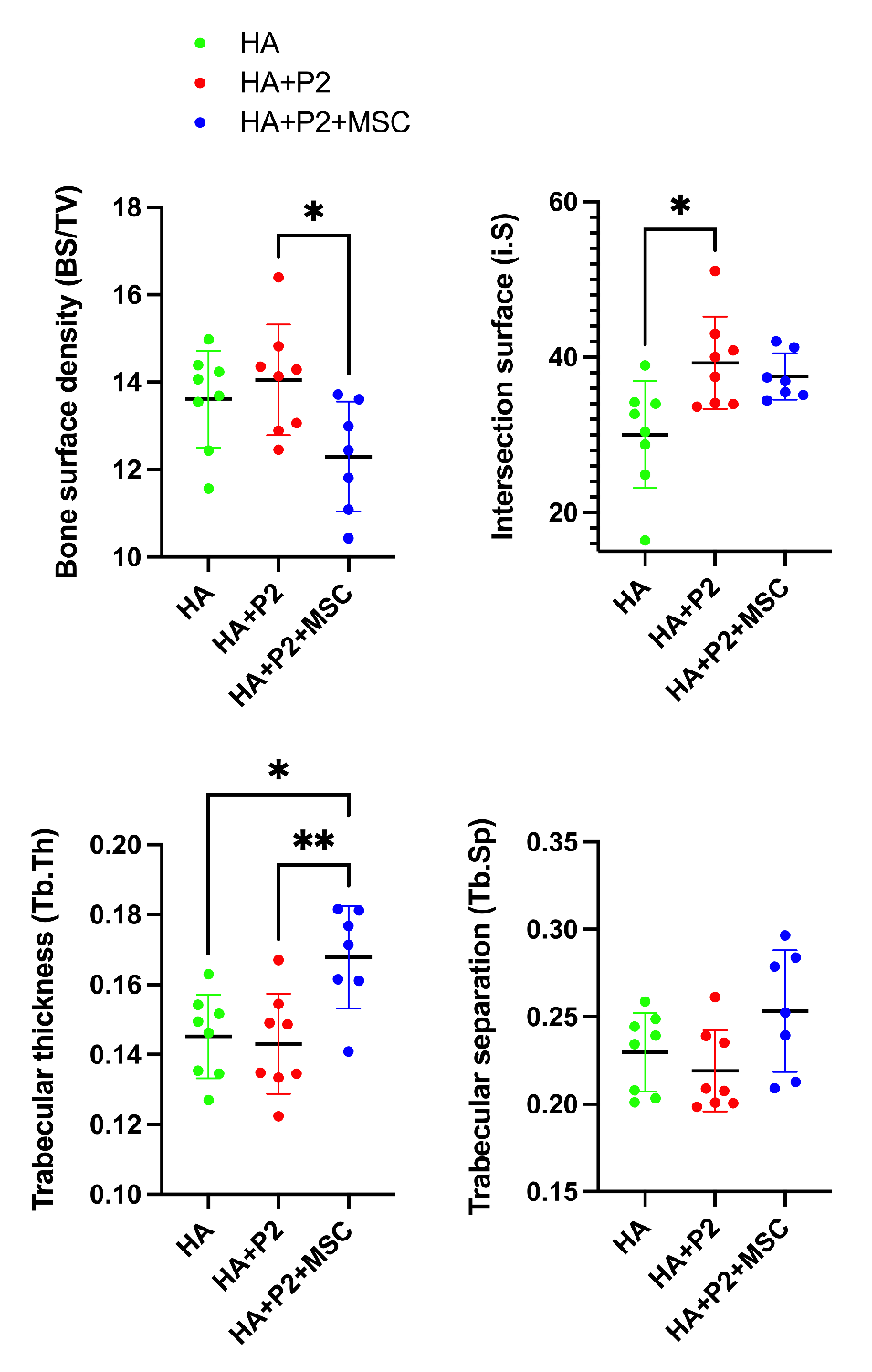
**

**Supp Figure 5.** Intrinsically disordered peptide 2 and mesenchymal stem cells (MSCs) enhance fracture healing in polytrauma. Quantitative µCT analysis of bone surface density (BS/TV), intersection surface (i.S), trabecular thickness (Tb.Th), and trabecular separation (Tb.Sp) in the three groups of hyaluronic acid-based hydrogel (HA), HA+P2, and HA+P2+MSC after three weeks of healing. Data are mean +/- standard deviation. N=8.

**
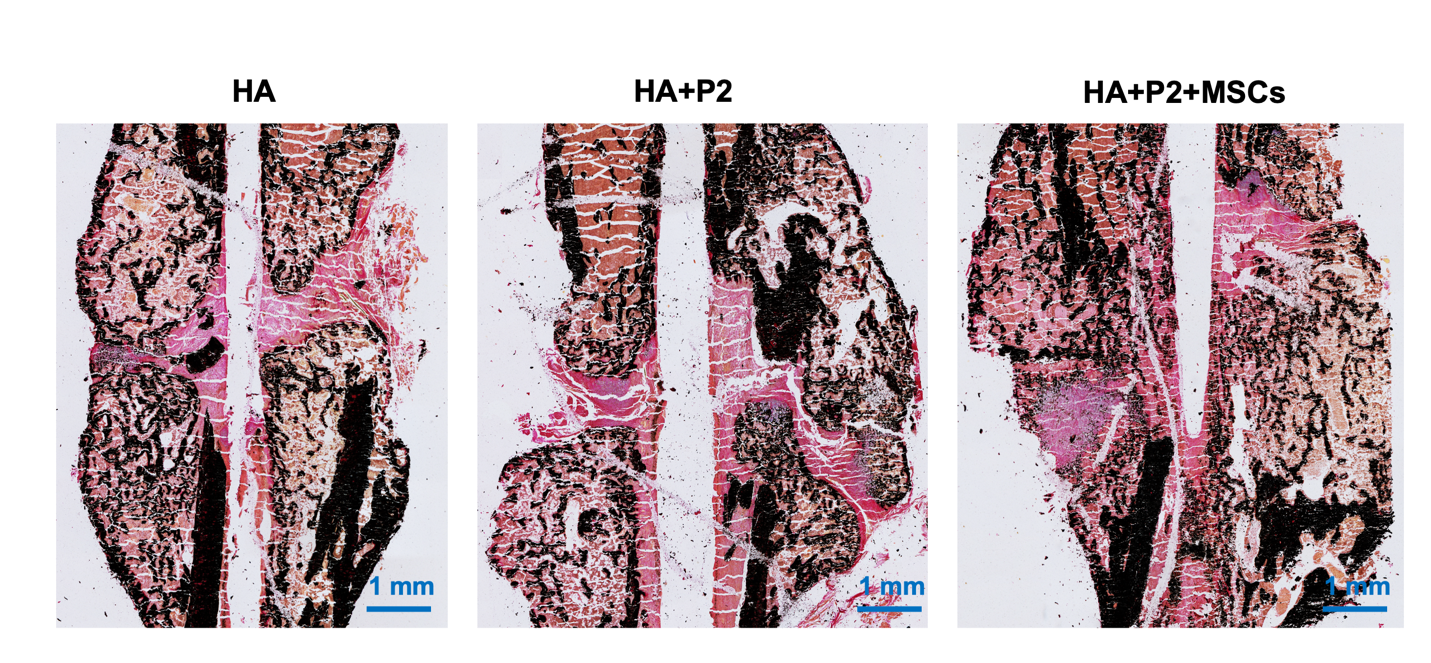
**

**Supp Figure 6.** Representative images of studying mineralized/non-mineralized balance of calcified samples using Von Kossa/Van Gieson histology staining to confirm our Movat Pentachrome results. N=8, scale bar= 1 mm.

**
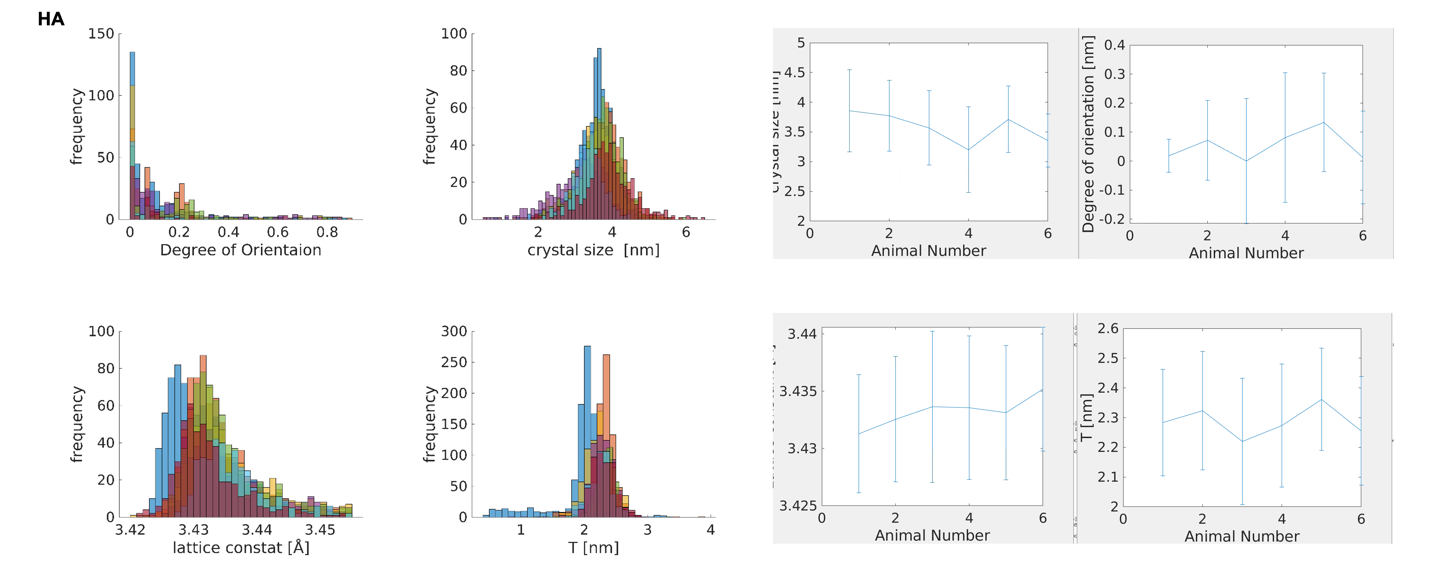
Supp Figure 7.** SAXS/XRD analysis of bone tissue from the Hydrogel-only group. Representative SAXS/XRD profiles of femoral defect sites treated with hydrogel alone, collected at 3 weeks post-injury. The SAXS data reflect relatively small mineral platelet thickness and limited orientation, while the XRD patterns show low crystallinity, indicating immature and disorganized mineralization. These findings suggest that hydrogel alone supports only minimal bone regeneration under polytrauma conditions. N=8.

**
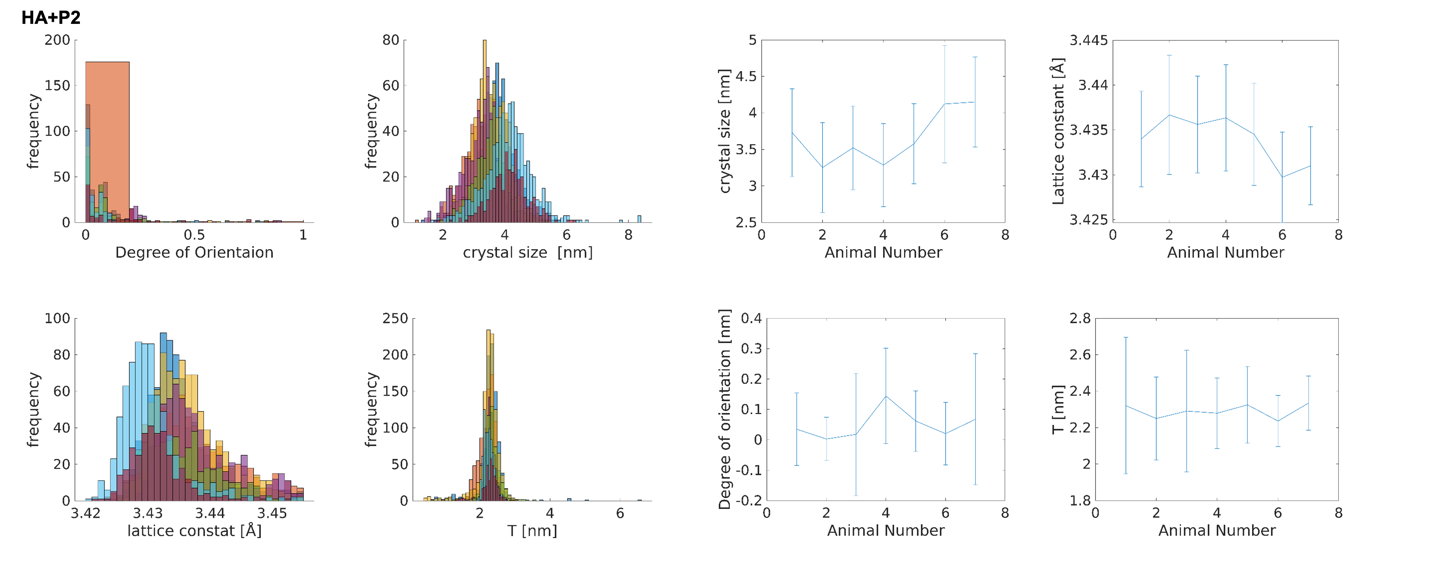
**

**Supp Figure 8.** **SAXS/XRD analysis of bone tissue from the** hyaluronic acid-based hydrogels containing P2 (HA+P2)**.** Profiles from the HA+P2 show moderately increased mineral platelet thickness and better alignment of hydroxyapatite crystals compared to hydrogel alone. XRD analysis demonstrates improved, yet still suboptimal, crystallinity. These data support the role of P2 in promoting early mineral organization and osteogenesis. N=8..

**
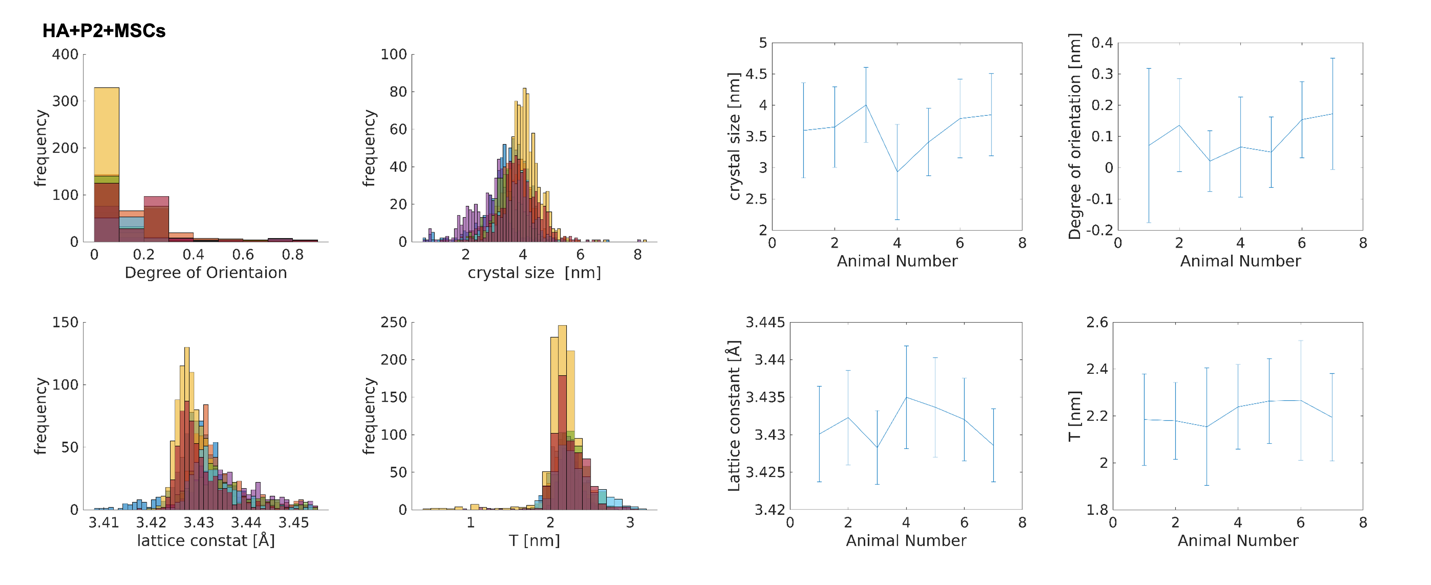
**

**Supp Figure 9.** SAXS/XRD analysis of bone tissue from the hydrogel with P2 and mesenchymal stem cells (HA+P2+MSCs) group. The SAXS analysis reveals the groups' greatest mineral platelet thickness and alignment, suggesting mature and organized bone matrix development. XRD profiles show sharper and more intense peaks, indicating enhanced mineral crystallinity. These findings confirm that the combination of P2 and MSCs synergistically improves bone tissue quality and regeneration. N=8.

**
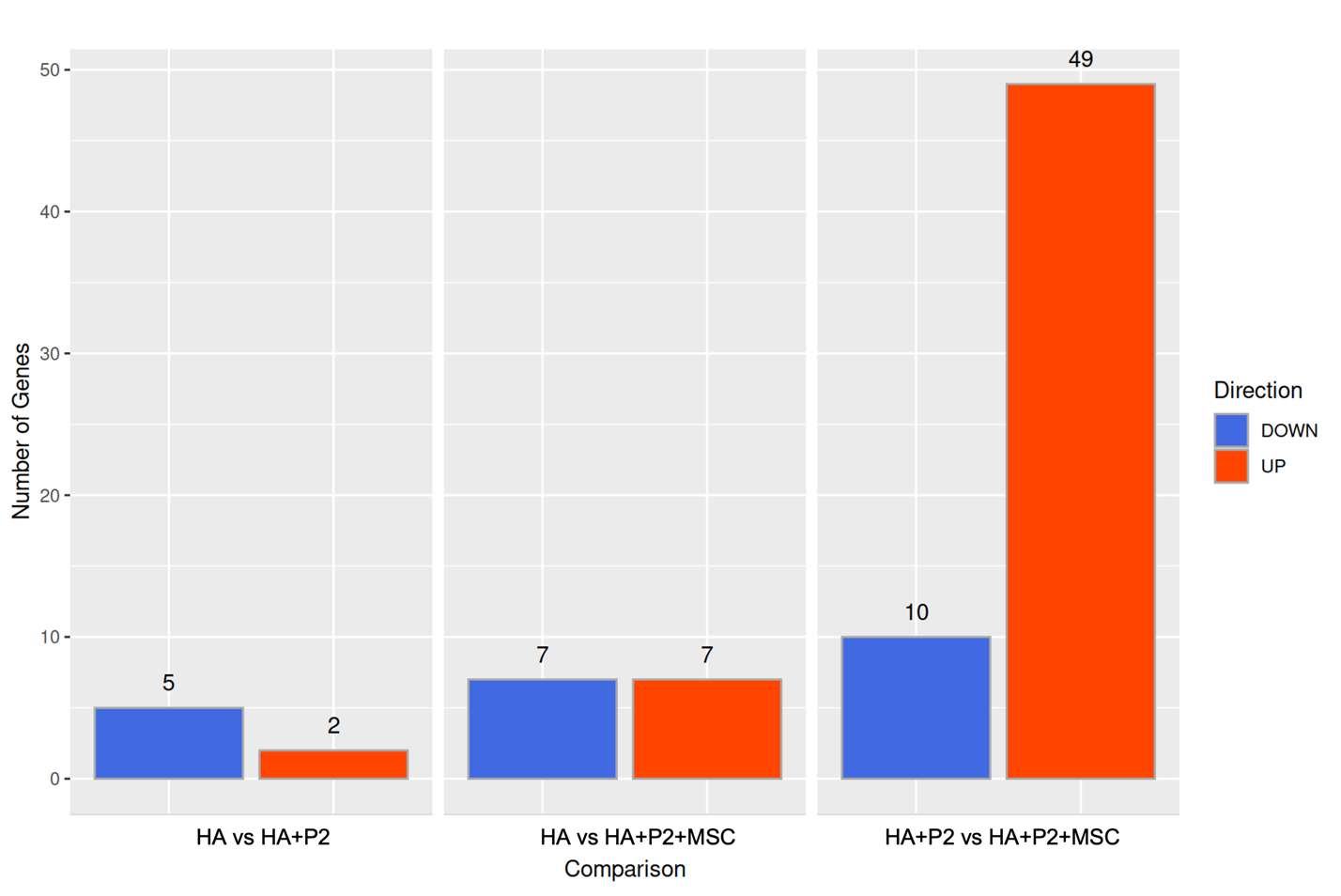
**

**Supp Figure 10.** Number of significant (adjusted p-value < 0.05) differentially expressed genes distinguished by direction of expression change. Up-regulated genes were more highly expressed by the second group listed in the comparison. N=4.

**Supp Table 1.** Differential expression analysis highlighting significantly upregulated and downregulated genes in hyaluronic acid-based hydrogels (HA) vs. HA+P2 groups. N=4.

**
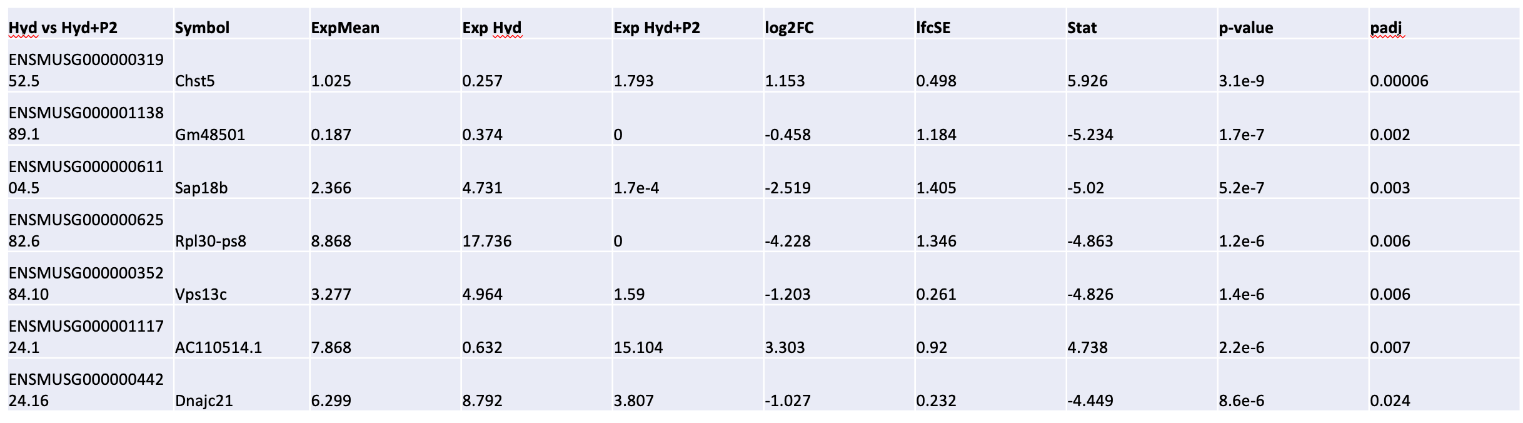
**

**Supp Table 2.** Gene ontology (GO) analysis identifying biological processes and pathways enriched in hyaluronic acid-based hydrogels (HA) vs. HA+P2 groups. N=4.

**
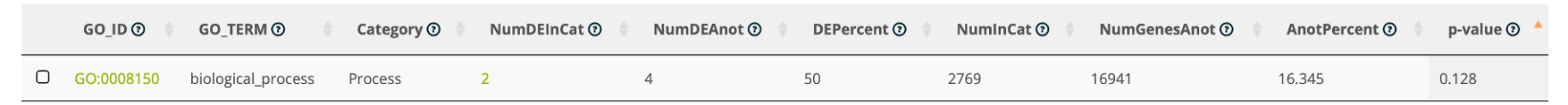
**

**Supp Table 3.** Differential expression analysis highlighting significantly upregulated and downregulated genes in hyaluronic acid-based hydrogels (HA) vs. HA+P2+MSC groups. N=4.

**
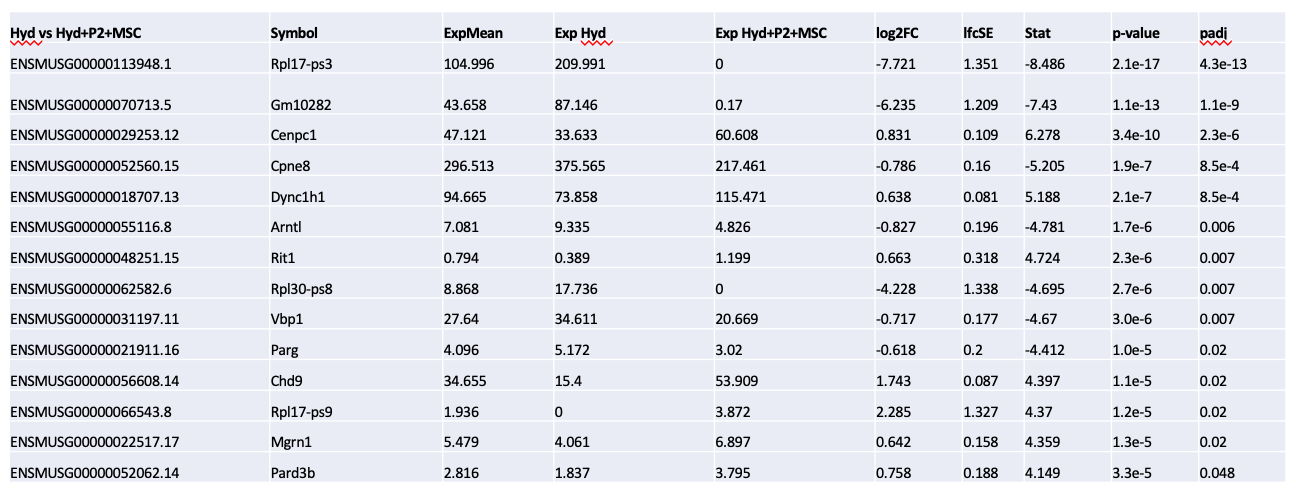
**

**Supp Table 4.** Gene ontology (GO) analysis identifying biological processes and pathways enriched in hyaluronic acid-based hydrogels (HA) vs. HA+P2+MSC groups. N=4.

**
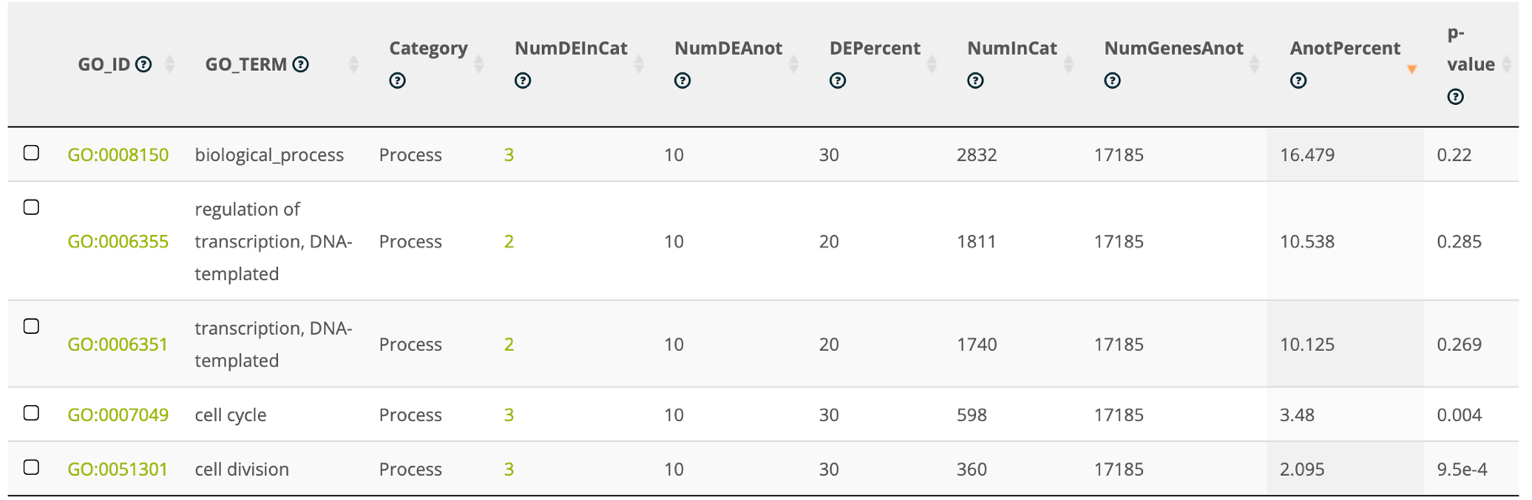
**

**Supp Table 5.** Differential expression analysis highlighting significantly upregulated and downregulated genes in HA+P2 vs HA+P2+MSC. N=4.

**
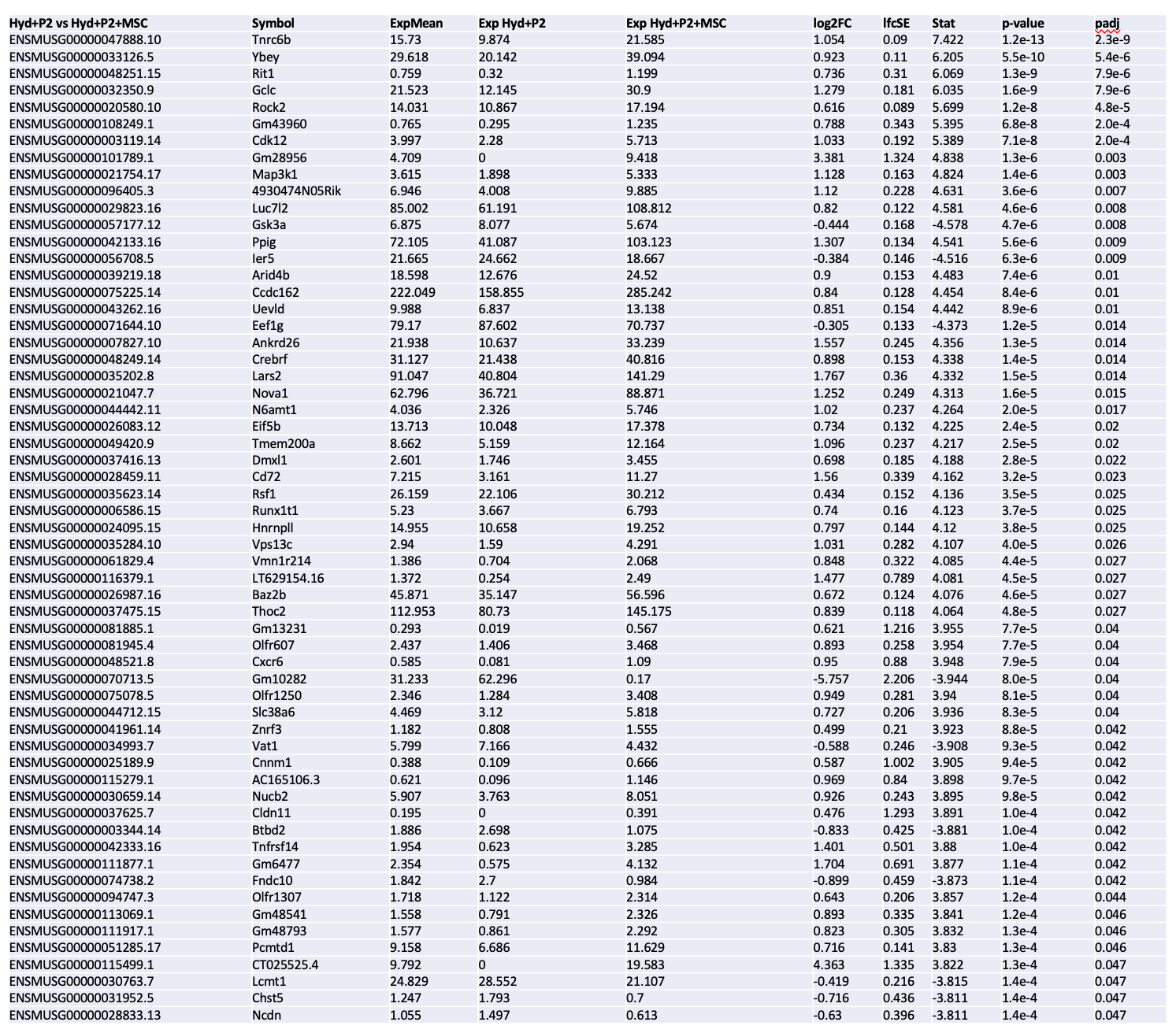
**

**Supp Table 6.** Gene ontology (GO) analysis identifying biological processes and pathways enriched in HA+P2 vs. HA+P2+MSC groups. N=4.

**
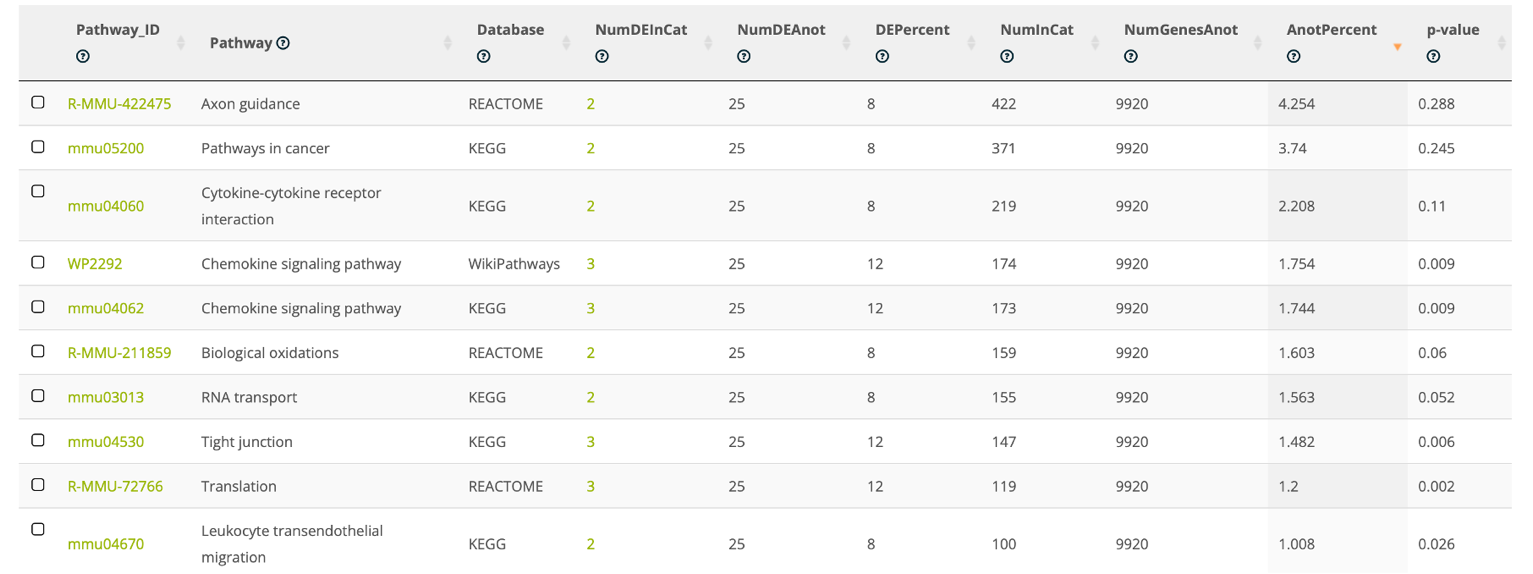
**

**References:**

1. Kim B, Peppas NA. In vitro release behavior and stability of insulin in complexation hydrogels as oral drug delivery carriers. International journal of pharmaceutics. 2003;266(1-2):29–37.
